# Sonosynthetic Cyanobacteria Oxygenation for Self‐Enhanced Tumor‐Specific Treatment

**DOI:** 10.1002/advs.202400251

**Published:** 2024-06-12

**Authors:** Zhenyu Yang, Xiu Shen, Junyi Jin, Xiaoyan Jiang, Wenqi Pan, Chenyao Wu, Dehong Yu, Ping Li, Wei Feng, Yu Chen

**Affiliations:** ^1^ Materdicine Lab School of Life Sciences Shanghai University Shanghai 200444 P. R. China; ^2^ School of Environmental and Chemical Engineering Shanghai University Shanghai 200444 P. R. China; ^3^ School of Medicine Shanghai University Shanghai 200444 P. R. China; ^4^ Department of Ultrasound Shanghai General Hospital Shanghai Jiao Tong University School of Medicine Shanghai 200080 P. R. China; ^5^ Oujiang Laboratory (Zhejiang Lab for Regenerative Medicine, Vision, and Brain Health) Wenzhou Institute of Shanghai University Wenzhou Zhejiang 325088 P. R. China

**Keywords:** cyanobacteria, sonoafterglow, sonodynamic therapy ferroptosis, sonosynthesis

## Abstract

Photosynthesis, essential for life on earth, sustains diverse processes by providing nutrition in plants and microorganisms. Especially, photosynthesis is increasingly applied in disease treatments, but its efficacy is substantially limited by the well‐known low penetration depth of external light. Here, ultrasound‐mediated photosynthesis is reported for enhanced sonodynamic tumor therapy using organic sonoafterglow (ultrasound‐induced afterglow) nanoparticles combined with cyanobacteria, demonstrating the proof‐of‐concept sonosynthesis (sonoafterglow‐induced photosynthesis) in cancer therapy. Chlorin e6, a typical small‐molecule chlorine, is formulated into nanoparticles to stimulate cyanobacteria for sonosynthesis, which serves three roles, i.e., overcoming the tissue‐penetration limitations of external light sources, reducing hypoxia, and acting as a sonosensitizer for in vivo tumor suppression. Furthermore, sonosynthetic oxygenation suppresses the expression of hypoxia‐inducible factor 1α, leading to reduced stability of downstream SLC7A11 mRNA, which results in glutathione depletion and inactivation of glutathione peroxidase 4, thereby inducing ferroptosis of cancer cells. This study not only broadens the scope of microbial nanomedicine but also offers a distinct direction for sonosynthesis.

## Graphical abstract

Ultrasound‐mediated photosynthesis for enhanced sonodynamic tumor therapy using organic sonoafterglow (ultrasound‐induced afterglow) nanoparticles combine with cyanobacteria, demonstrating the proof‐of‐concept sonosynthesis (sonoafterglow‐induced photosynthesis) in cancer therapy (Created with BioRender.com).

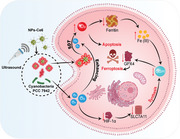

## 1 Introduction

Photosynthesis, the fundamental biological process on earth, harnesses solar energy to convert carbon dioxide and water into chemical energy and oxygen.^[^ ^1^ ^]^ It is crucial in addressing contemporary challenges in food, energy, and environmental resources.^[^ ^2^ ^]^ Various artificial photosynthetic systems have been constructed and employed for green energy.^[^ ^3^ ^]^ Even a plant‐derived photosynthetic system was transferred to mammalian cells to cure osteoarthritis.^[^ ^4^ ^]^ Beyond plants, microbial photosynthesis is vital in nature and has been innovatively integrated into biomedical applications,^[^ ^5^ ^]^ particularly for oxygen‐dependent disease treatments.^[^ ^6^ ^]^ The photocatalytic oxygen production capacity and biocompatibility make photosynthetic microorganisms suitable for treating hypoxia‐related diseases, including tumor therapy,^[^ ^7^ ^]^ wound healing,^[^ ^8^ ^]^ tissue engineering,^[^ ^9^ ^]^ and ischemic myocardial injury treatment.^[^ ^10^ ^]^ However, the effectiveness of photosynthesis in medical applications is limited by the poor tissue penetration of light sources and interference from endogenous tissue substances, hindering activation in deep tissues and reducing the therapeutic effect.^[^ ^11^ ^]^ Moreover, prolonged light irradiation or increased power densities pose potential issues, challenging the clinical translation of photostimulated microbial therapy.^[^ ^12^ ^]^ Therefore, optimizing the activation source is highly crucial for effective photosynthetic oxygen therapy.

The development of nanotechnology, particularly light conversion nanomaterials such as upconversion nanoparticles (UCNPs),^[^ ^13^ ^]^ nanoscintillators,^[^ ^14^ ^]^ and persistent luminescence nanoparticles (PLNPs),^[^ ^15^ ^]^ has significantly advanced therapy based on light. UCNPs and nanoscintillators efficiently convert near‐infrared (NIR) light and X‐ray into ultraviolet (UV) or visible light, offering deeper tissue penetration.^[^ ^11^ ^]^ PLNPs, stimulated by external light sources such as UV light and X‐rays maintain prolonged afterglow luminescence, potentially shorting irradiation time and minimizing tissue photodamage.^[^ ^16^ ^]^ Additionally, surgically implanted photonic patches address light source limitations through direct irradiation.^[^ ^17^ ^]^ Despite these advances, challenges such as poor biodegradability, low light conversion efficiency, and inefficient targeting limit their potential. UCNPs suffer from non‐degradability and relatively low light conversion efficiencies.^[^ ^18^ ^]^ Nanoscintillators require constant X‐ray irradiation, posing side effects on patients. PLNPs still need frequent charging from external energy sources.^[^ ^19^ ^]^ Photonic patches restrict patient mobility and pose biosafety concerns for long‐term implantation.^[^ ^20^ ^]^ Consequently, there is a critical need for a treatment strategy with high penetration capabilities to enhance microbial therapy efficacy. Ultrasound (US) emerges as a promising alternative, offering superior penetration and targetability than light, especially in vivo.^[^ ^21^ ^]^ However, the sonostimulated microbial photosynthesis in disease treatment remains underexplored.

**Figure 1:**
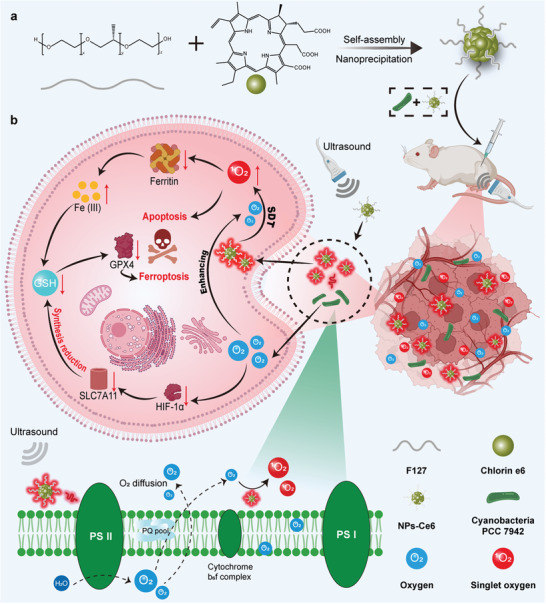
Schematic illustration of sonoafterglow‐enhanced photosynthetic oxygenation to boost SDT. a) Diagram of the fabrication process of NPs‐Ce6. b) The antitumor mechanisms of sonosynthesis‐enhanced SDT lead to ferroptosis and apoptosis (Created with https://www.BioRender.com).

In this study, we develop a sonoafterglow‐induced cyanobacterial photosynthesis‐based sonodynamic therapy (SDT) platform and demonstrate a proof‐of‐concept sonosynthesis (sonoafterglow‐induced photosynthesis) by combining *Synechococcus elongatus* PCC 7942 (PCC) with sonoafterglow Ce6 nanoparticles (NPs‐Ce6) (**Figure** **1**). This platform mitigates tumor hypoxia using the sustained afterglow of NPs‐Ce6 to stimulate PCC oxygen production while enhancing SDT by singlet oxygen (^1^O_2_) generation under US irradiation. Tumor hypoxia contributes significantly to resistance against ferroptosis,^[^ ^22^ ^]^ due to the stabilization of solute carrier family 7 member 11 (SLC7A11) mRNA, a gene vital for cystine transport and glutathione (GSH) synthesis,^[^ ^23^ ^]^ facilitated by hypoxia‐inducible factor 1α (HIF‐1α).^[^ ^24^ ^]^ Under US irradiation, NPs‐Ce6 emits continuous red light, inducing prolonged PCC photosynthesis, alleviating tumor hypoxia, and improving SDT. The reduced hypoxia leads to lower HIF‐1α levels, decreasing SLC7A11 subunit activity, impeding GSH synthesis, and reducing tumor cell resistance to ferroptosis. Additionally, the SDT‐produced ^1^O_2_ disrupts ferritin, releasing Fe^3+^ ions, further depleting GSH and intensifying tumor cell ferroptosis. This sonosynthesis‐based therapeutic strategy has the potential to overcome the deep hypoxic disease environment by enhancing oxygenation, thereby improving the effectiveness of treatments such as SDT.

## 2 Results and Discussion

### 2.1 Synthesis and Characterization of NPs‐Ce6

**Figure 2:**
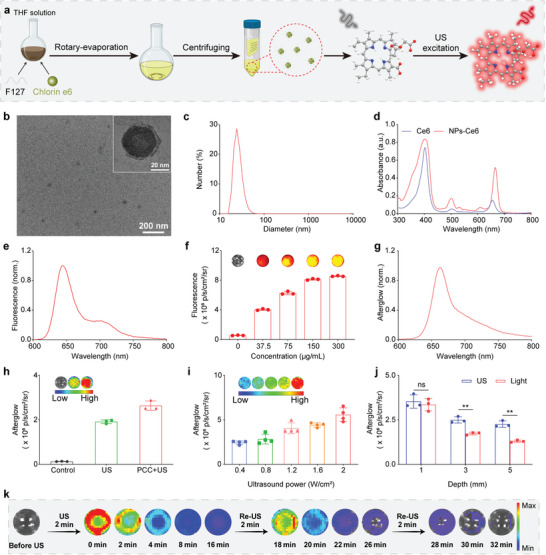
Synthesis and characterization of NPs‐Ce6. a) Schematic illustration showing the synthesis of NPs‐Ce6 (Created with BioRender.com). b) TEM images of NPs‐Ce6. c) Size distribution of NPs‐Ce6 in PBS. d) UV‐vis absorbance spectra of Ce6 in THF and NPs‐Ce6 in PBS. e) Normalized fluorescence spectrum of NPs‐Ce6 in PBS under excitation at 400 nm. f) Concentration‐dependent fluorescence intensity of NPs‐Ce6 (*n* = 3). g) Normalized afterglow luminescence spectrum of NPs‐Ce6 after US irradiation. h) Sonoafterglow intensity of NPs‐Ce6 after different treatment (*n* = 3). i) US irradiation power‐dependent sonoafterglow intensity of NPs‐Ce6 (*n* = 4). j) Afterglow intensity of NPs‐Ce6 after US or light irradiation at different chicken breast tissue thicknesses. (Ultrasound parameters: 1.0 MHz, 50% duty, 1.5 W cm^−2^, 60 s. Laser parameters: 660 nm, 1.5 W cm^−2^, 60 s. *n* = 3). NPs‐Ce6 concentration: 500 µg mL^−1^, PCC concentration: 1 × 10^6^ cell mL^−1^. Statistical differences of *p* values were determined by One‐way ANOVA. k) Sonoafterglow luminescence images of NPs‐Ce6 after US irradiation for 2 min and re‐activation for another 2 min. Data are presented as mean values ± SD. ^**^ *p* < 0.01.

A bottom‐up nanoprecipitation technique was employed to synthesize the NPs‐Ce6 from the homogeneously dissolved tetrahydrofuran (THF) solution of Ce6. Briefly, PEG‐b‐PPG‐b‐PEG and Ce6 were dissolved into a THF solution. The mixture was then injected into ultrapure water (45 mL) under magnetic stirring. Then the THF was evaporated with a rotary evaporator. Finally, the resulting solution of NPs‐Ce6 was received by ultracentrifugation with 30 K centrifugal filter units and the concentrated solution of NPs‐Ce6 was diluted with 1 × PBS buffer. The obtained nanoparticle dispersion was stored in the dark at 4 °C (**Figure** **2**a). NPs‐Ce6 showed spherical morphology with a hydrodynamic size of 24.6 ± 3.6 nm (Figure 2b,c). The ultraviolet‐visible (UV‐vis) absorbance spectrum of the Ce6 exhibited a prominent absorption peak at ≈400 nm, and this distinctive absorption peak was likewise observed in NPs‐Ce6 (Figure 2d). The emission of NPs‐Ce6 excited at 400 nm was tracked between 610 and 750 nm (Figure 2e), in which the fluorescence intensity was dependent on the concentration (Figure 2f). Notably, the examination of the afterglow spectrum of NPs‐Ce6 following US irradiation revealed the presence of a prominent peak at 680 nm (Figure 2g). Importantly, it was observed that NPs‐Ce6 could effectively produce afterglow luminescence under US stimulation (Figure 2h), confirming the sonoafterglow ability of NPs‐Ce6. Moreover, after co‐incubation PCC, the afterglow luminescence intensity of NPs‐Ce6 after US activation further increased compared with only NPs‐Ce6. Furthermore, the sonoafterglow intensity immediately recovered upon re‐exposure to the US (three rounds) after a continuous decline (Figure 2k; Figure S1a, Supporting Information). Additionally, the sonoafterglow intensity closely correlated with the duration and power of US irradiation (Figure 2i; Figure S1b, Supporting Information), in which the sonoafterglow intensity increased with the incremental US time and power density. To investigate the advantage of US penetration depth, NPs‐Ce6 dispersion was covered with chicken breast tissues, followed by US or laser irradiation (Figure S3, Supporting Information). Due to the superior penetration ability of US, the intensity of sonoafterglow was notably higher than that of laser‐mediated photoafterglow when penetrating chicken breast tissues at the thicknesses of 3 and 5 mm (Figure 2j), demonstrating the effective activation of sonoafterglow in deep tissues for the enhanced therapeutic potential.

To elucidate the molecular mechanism underlying the sonoafterglow phenomenon induced by Ce6 after US irradiation, the structural alterations of Ce6 were analyzed. The absorption spectra detailed in Figure S2a (Supporting Information) reveal a significant decrease in the primary absorption peak of Ce6 ≈ 400 nm following US irradiation, suggesting substantial Ce6 decomposition. Further examination through mass spectrometry to assess the Ce6 changes before and after US treatment indicated the emergence of a new compound, exhibiting a molecular ion peak at m/z = 363.90 ([M + H]+). Correlating with existing literature, the entity was identified as Ce6‐F (Figure S2c, Supporting Information).^[^ ^25^ ^]^ These findings lead to the proposition of sonoafterglow mechanism for Ce6: energy absorbed by Ce6 is transferred to oxygen, facilitating the ^1^O_2_ production. Subsequently, ^1^O_2_ engages in a [*π*2 + *π*2] cycloaddition with the vinylene linkage (C═C) of Ce6, generating a Ce6‐dioxetane intermediate. The eventual breakdown of this intermediate is responsible for the sonoafterglow luminescence. Given the similarity between the sonoafterglow and Ce6 fluorescence, it is inferred that the spontaneous disintegration of the Ce6‐dioxetane intermediate channels the requisite energy for elevating Ce6 to an excited state (Ce6*), which emits sonoafterglow luminescence upon returning to the ground state. Meanwhile, Ce6‐F formation occurs (Figure S2d, Supporting Information).^[^ ^26^ ^]^

### 2.2 Characterization of Cyanobacteria

**Figure 3:**
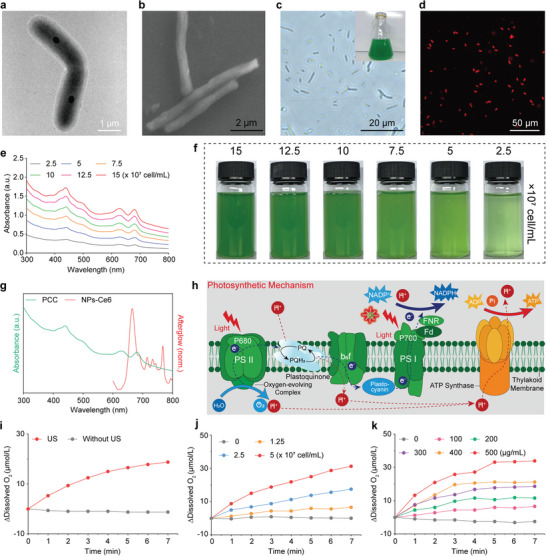
Sonoafterglow‐mediated photosynthetic oxygenation. a) TEM image of PCC. b) Scanning electron micrograph (SEM) image of PCC. c) Optical microscope image of PCC (inset, digital photo of PCC during preparation). d) Fluorescence microscope image of PCC. e) UV‐vis absorbance spectra and f) the corresponding optical images of PCC at different concentrations. g) UV‐vis absorbance spectrum of PCC and sonoafterglow luminescence spectrum of NPs‐Ce6. h) Schematic illustration showing the photosynthetic mechanism of PCC (Created with BioRender.com). i) Dissolved oxygen levels of PCC with NPs‐Ce6 after different treatments. j) Concentration‐dependent oxygenation of PCC treated with NPs‐Ce6 under US irradiation (NPs‐Ce6 500 µg mL^−1^, 2 W cm^−2^, 3 min). k) Concentration‐dependent oxygenation of NPs‐Ce6 under US irradiation with PCC (PCC 5 × 10^7^ cell mL^−1^, 2 W cm^−2^, 3 min).

PCC displayed a distinctive rod‐shaped morphology with micrometer size (**Figure** **3**a,b). In addition, the surface of PCC exhibited a negative charge attribute (Figure S4, Supporting Information). During their mid‐exponential phase, PCC was cultured in BG11 medium and continuously supplemented with fresh medium to promote healthy growth, in which the characteristic green coloration of the culture medium was attributed to the optical density at 730 nm (OD730) in the ranges of 0.4 to 0.7 (Figure 3c, inset). The red autofluorescence, attributed to phycocyanin fluorescence in PCC, was confirmed using a confocal laser scanning microscope (CLSM) (Figure 3d; Figure S5, Supporting Information). The broad absorption spectrum, with prominent peaks at 440, 630, and 681 nm, was attributed to the presence of chlorophyll a. It was important to note that the absorption intensity showed a positive correlation with the concentration of PCC (Figure 3e,f). To determine the amount of PCC, a standard curve fcor PCC concentration was established by measuring OD730 (Figure S6, Supporting Information). The overlap between the emission spectrum of NPs‐Ce6 and the absorption spectrum of PCC is a crucial factor in photonic activation. The absorption peak at 680 nm, attributed to chlorophyll a, as shown in Figure 3g, confirmed the effective activation of photosynthesis under red light irradiation, which guaranteed the oxygenation by the afterglow of NPs‐Ce6 (Figure 3h). Subsequently, to assess the oxygen evolution capacity of PCC, a portable dissolved oxygen electrode was utilized. At first, we excluded the impact of varying NPs‐Ce6 concentrations on dissolved oxygen levels after US irradiation (Figure S7, Supporting Information). After that, the effect of NPs‐Ce6 on the oxygen production of PCC was examined (Figure 3i). It was found that NPs‐Ce6, after US irradiation, effectively enhanced the oxygen production of PCC, as indicated by the increased dissolved oxygen levels. In contrast, NPs‐Ce6 without US irradiation not only failed to promote dissolved oxygen production but also reduced it due to the respiration of PCC. This confirmed that sonoafterglow could effectively enhance the oxygen‐producing capacity of PCC. Moreover, both NPs‐Ce6 and PCC exhibited a concentration‐dependent relationship with the generation of dissolved oxygen (Figure 3j,k). To verify the viability of PCC, it was cultured with PBS (pH 6.4), 1640 medium, and different concentrations of NPs‐Ce6. Remarkably, even in the presence of a high concentration of NPs‐Ce6 at 500 µg mL^−1^, PCC maintained its proliferative ability after US stimulation during a 7‐day observation period (Figure S8, Supporting Information), suggesting that NPs‐Ce6 and ^1^O_2_ have no obvious toxicity on PCC growth. Furthermore, the oxygen‐generating capacity of PCC was evaluated after 7 days (Figure S9, Supporting Information). The photosynthetic oxygen production of PCC remained largely consistent with its performance in BG11 medium, establishing a solid foundation for the following in vitro and in vivo experiments.

### 2.3 Sonosynthetic Oxygenation for Self‐Enhanced SDT

**Figure 4:**
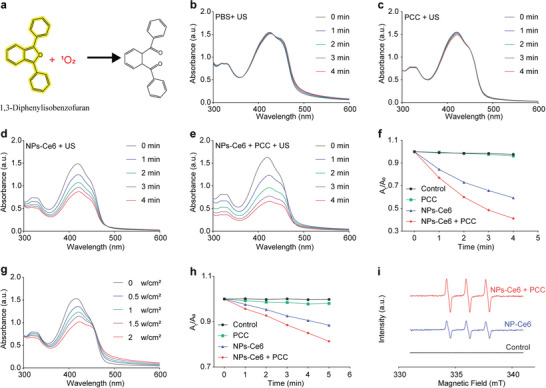
Sonodynamic performance of NPs‐Ce6 + PCC. a) DPBF structure and color change before and after interacting with ^1^O_2_. Time‐dependent ^1^O_2_ generation of b) PBS, c) PCC, d) NPs‐Ce6, and e) NPs‐Ce6 + PCC under US irradiation (1.5 W cm^−2^) for 4 min detected with the DPBF probe. f) Quantitative analysis of DPBF oxidation after different treatments. g) Power‐dependent ^1^O_2_ generation of NPs‐Ce6 + PCC under US treatment with the DPBF probe (0, 0.5, 1, 1.5, and 2 W cm^−2^, 2 min). h) Quantitative analysis of ABDA oxidation after different treatments (1.5 W cm^−2^). i) ESR spectra using TEMP as trapper after different treatments. (1.5 W cm^−2^, 2 min). NPs‐Ce6 concentration: 500 µg mL^−1^, PCC concentration: 1 × 10^6^ cell mL^−1^.

Benefiting from the ability of NPs‐Ce6 to promote oxygen production in PCC through photosynthesis via sonoafterglow, we hypothesized that sonosynthesis could increase the ROS production of NPs‐Ce6. 1,3‐diphenylisobenofuran (DPBF), a yellow indicator that fades after reacting with ^1^O_2_, was used to examine the enhancement of SDT through sonoafterglow‐induced oxygen production (**Figure** **4**a). First, the potential adsorption effects of NPs‐Ce6 and PCC on characteristic peak reduction were eliminated (Figure S10, Supporting Information). Under US irradiation, a significant reduction in characteristic peaks was observed over time in both the NPs‐Ce6 and NPs‐Ce6 + PCC groups compared to that in PBS and PCC groups (Figure 4b–e), indicating that the PCC group does not generate ^1^O_2_, while both NPs‐Ce6 and NPs‐Ce6 + PCC groups exhibited a time‐dependent ^1^O_2_ production. Furthermore, under the same US conditions, the NPs‐Ce6 + PCC group exhibited a higher ^1^O_2_ yield than the NPs‐Ce6 group alone, suggesting that sonoafterglow‐enhanced oxygen production led to increased ROS production (Figure 4f). When subjected to varying US power density levels, the characteristic peaks in the NP‐Ce6 + PCC group were diminished with the power densities increased (Figure 4g). These results were further confirmed by the 9,10‐anthracenediyl‐bis‐(methylene)dimalonic acid (ABDA) indicator, which showed similar outcomes to DPBF and provided additional evidence for sonoafterglow‐augmented oxygen production in PCC, resulting in enhanced ^1^O_2_ production (Figure 4h; Figure S11, Supporting Information). Additionally, electron spin resonance (ESR) spectroscopy was utilized to detect and identify the ^1^O_2_ generation, using 2,2,6,6‐tetramethyl‐4‐piperidone (TEMP) as a trapping agent. The pronounced ^1^O_2_‐induced ESR signals were observed in the ESR spectrum following NPs‐Ce6 treatment under US irradiation (Figure 4i), evidenced by the appearance of a characteristic 1:1:1 signal. Compared to the NPs‐Ce6 + US group, the NPs‐Ce6 + PCC + US group exhibited a stronger ESR signal, indicating higher ^1^O_2_ yielded due to photosynthetic oxygenation stimulated by sonoafterglow, thereby effectively enhancing the sonodynamic effect.

### 2.4 In Vitro Therapeutic Effect

**Figure 5:**
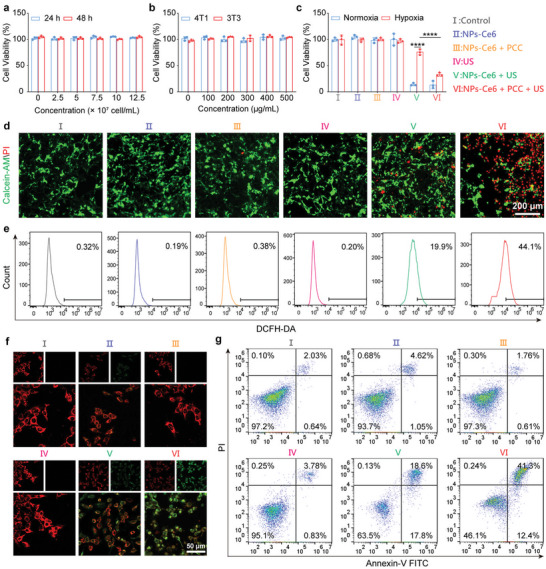
In vitro therapeutic effect. a) Relative viabilities of 4T1 cancer cells after treatment with cyanobacteria at different concentrations for 24 and 48 h (*n* = 3). b) Relative viabilities of 4T1 cells and 3T3 cells after treatment with NPs‐Ce6 at different concentrations for 24 h (*n* = 3). c) Relative viabilities of 4T1 cancer cells after different treatments (*n* = 3). d) Fluorescence images of Calcein‐AM/PI co‐staining 4T1 cells. e) flow cytometry analysis of 4T1 cells staining with DCFH‐DA. f) CLSM of 4T1 cells staining with JC‐1 probe, and g) flow cytometry analysis of 4T1 cells staining with fluorescein‐annexin V‐FITC and PI after indicated treatments under hypoxic conditions. NPs‐Ce6 concentration: 400 µg mL^−1^, PCC concentration: 5 × 10^7^ cell mL^−1^ 1.2 W cm^−2^, 5 min. Data are presented as mean values ± SD. Statistical differences in *p* values were determined by One‐way ANOVA. ^****^ *p* < 0.0001.

Inspired by the excellent sonoafterglow‐mediated oxygen generation and sonodynamic capabilities, the in vitro SDT performance was further evaluated at the cellular level. Initially, the cytocompatibility of PCC and NPs‐Ce6 was evaluated independently. The results of the Cell Counting Kit‐8 (CCK‐8) assay demonstrate that neither PCC nor NPs‐Ce6 display significant cytotoxicity after co‐incubation for 24 and 48 h, confirming their benign biocompatibility (**Figure** **5**a; Figure S12, Supporting Information). Furthermore, the cytotoxicity of NPs‐Ce6 remained negligible even up to 500 µg mL^−1^ when tested on murine mammary carcinoma 4T1 cells and murine embryonic fibroblast 3T3 cells (Figure 5b), reinforcing its high biosafety. Therefore, in subsequent in vitro experiments, 400 µg mL^−1^ NPs‐Ce6 and 5 × 10^7^ cell mL‐1PCC were applied. The cellular uptake was examined through CLSM (Figure S13, Supporting Information), which revealed the NPs‐Ce6 uptake in 4T1 cells after co‐incubation for 2 h with an increase over time. The sonodynamic effect on 4T1 cancer cells was subsequently evaluated (Figure 5c), which induced a notable decrease in cell viability under normoxia in the NPs‐Ce6 + US group, highlighting the critical role of oxygen in efficient SDT. Significantly, the cell viability in the NPs‐Ce6 + PCC group decreased substantially post‐US irradiation under hypoxic conditions, demonstrating that sonoafterglow could promote oxygen generation in PCC, thereby enhancing SDT efficacy. In addition, the prolonged US irradiation duration further decreased the cell survival rates (Figure S14, Supporting Information).

Calcein acetoxymethyl ester (Calcein‐AM) and propidium iodide (PI) co‐staining assay were utilized for evaluating the live and death cells of SDT effect (Figure 5d; Figure S15, Supporting Information). As expected, the NPs‐Ce6 + US group showed partial red fluorescence, whereas the NPs‐Ce6 + PCC + US group exhibited strong red fluorescence, aligning with the CCK‐8 assay results. Additionally, dichlorofluorescein diacetate (DCFH‐DA) was utilized to assess ROS production. Flow cytometry analysis and CLSM images of 4T1 tumor cells stained with DCFH‐DA revealed significant green fluorescence (Figure 5e; Figures S16,  S17, Supporting Information), indicating that the sonosynthetic oxygenation boosted ROS generation. ROS production is known to damage mitochondria, as evidenced by the decrease in mitochondrial membrane potential observed through the transition of potential‐sensitive dye JC‐1 from red to green fluorescence. Finally, apoptosis in 4T1 cancer cells was investigated using flow cytometry analysis with fluorescein isothiocyanate (FITC)‐labeled Annexin V and PI staining method (Figure 5f). Compared to the NPs‐Ce6 + US group showing a significant late apoptosis ratio at 18.6%, the addition of PCC led to a higher rate of apoptosis (late apoptosis ratio at 41.3%), confirming the enhanced SDT therapeutic performance.

### 2.5 In Vitro Therapeutic Mechanism

**Figure 6:**
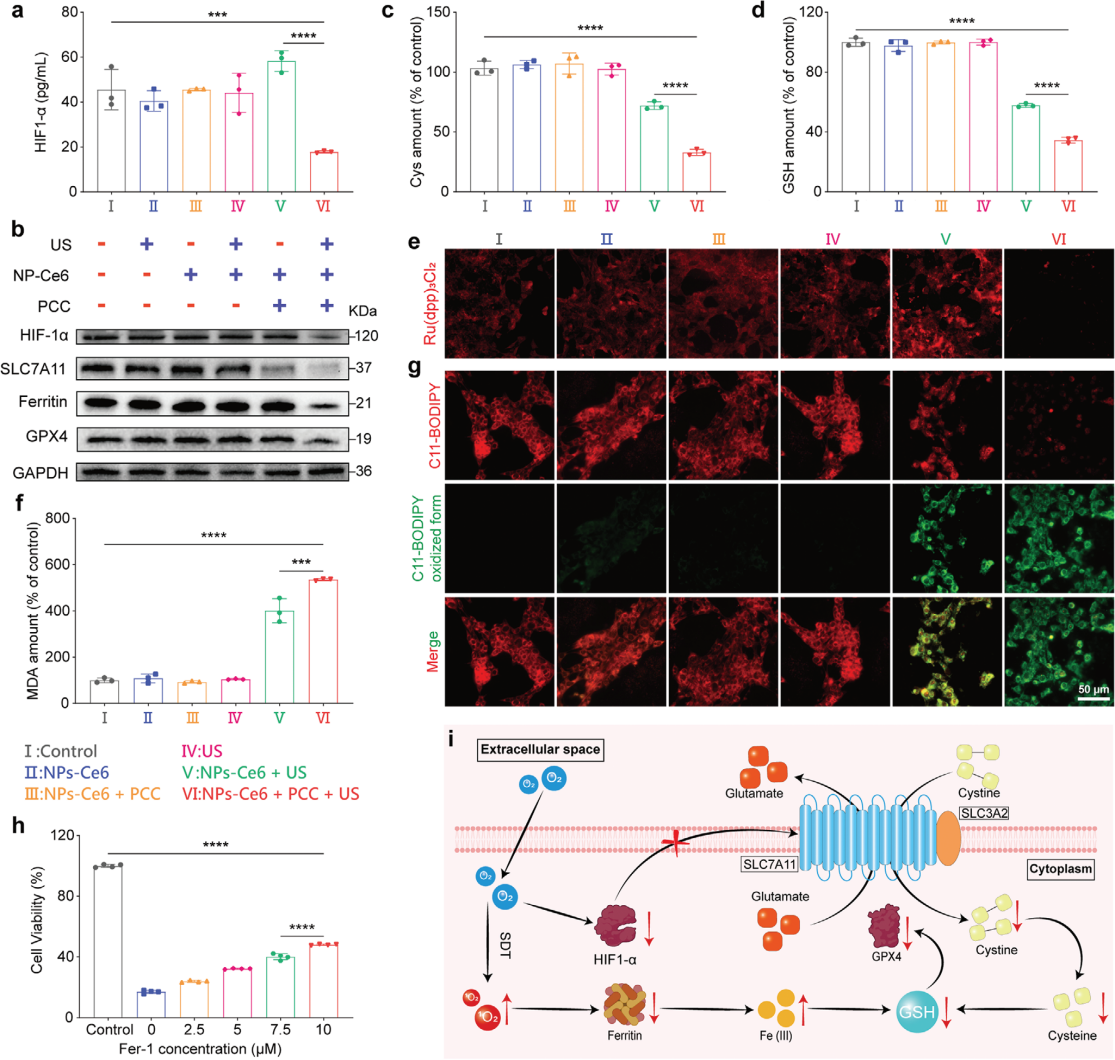
In vitro therapeutic mechanism. a) The concentration of HIF‐1α after different treatments in hypoxic conditions (*n* = 3). b) Western blot assay of HIF‐1α, SLC7A11, Ferritin, and GPX4 level in 4T1 cells treated after indicated treatments. c) Relative Cys content (*n* = 3) and d) relative GSH content in 4T1 cells after indicated treatments (*n* = 3). e) CLSM images of 4T1 cells staining with Ru(dpp)_3_Cl_2_ after indicated treatments. f) Relative MDA content in 4T1 cells after indicated treatments (*n* = 3). g) CLSM images of 4T1 cells staining with C11‐BODIPY^581/591^ dye after indicated treatments. h) Cell viability of 4T1 treated with NPs‐Ce6 + PCC+US after addition of different concentrations of Fer‐1 (*n* = 4). i) Schematic illustration showing that oxygen generation promotes SDT and ferroptosis (Created with BioRender.com, NPs‐Ce6 concentration: 400 µg mL^−1^, PCC concentration: 5 × 10^7^ cell mL^−1^, 1.2 W cm^−2^, 5 min). Data are presented as mean values ± SD. Statistical differences of *p* values were determined by One‐way ANOVA. ^***^ *p* < 0.001, ^****^ *p* < 0.0001.

NPs‐Ce6 in synergy with PCC had demonstrated remarkable cell‐killing efficiency under US radiation. We delved further into the mechanism underlying the cell‐killing effect. NPs‐Ce6, leveraging its sonoafterglow and sonodynamic properties, could enhance photosynthesis in PCC and alleviate tumor hypoxia, thereby augmenting SDT. Under hypoxic conditions, the NPs‐Ce6 + PCC + US group exhibited a significant decrease in HIF‐1α levels, demonstrating the effectiveness of sonosynthetic oxygen production in vitro (**Figure** **6**a). On the contrary, the NPs‐Ce6 + US group showed a marked increase in HIF‐1α levels, likely due to increased intracellular oxygen consumption during SDT. To further visualize the oxygen production, the same effect was observed using the intracellular hypoxia indicator [Ru(dpp)_3_]Cl_2_, which operates on an oxygen‐dependent fluorescence‐quenching mechanism. The weakest red fluorescence in the NPs‐Ce6 + PCC + US group indicated a reduction in hypoxia (Figure 6e). Moreover, western blot analysis indicated that, compared to other groups, the NPs‐Ce6 + PCC + US group led to the down‐regulation of HIF‐1α expression, inhibition of amino acid transporter SLC7A11 expression, and the destruction of ferritin, ultimately resulting in GPX4 inactivation in cancer cells (Figure 6b; Figure S18, Supporting Information).

Additionally, ROS facilitated the destruction of ferritin in cells, releasing endogenous iron ions and further depleting GSH, which indirectly decreased GPX4 expression. The cytoplasmic iron levels, a critical indicator of ferroptosis, were assessed using Calcein (CAL). Since iron chelates with CAL to quench its fluorescence, the fluorescence intensity inversely correlates with iron content. As expected, the signal was the weakest in the NPs‐Ce6 + PCC + US group, signifying the most extensive damage to ferritin and the highest iron ions release (Figure S19, Supporting Information). Consequently, following the decrease in SLC7A11 expression, cellular levels of cysteine (Cys) and GSH were further measured (Figure 6c,d). Cys and GSH levels were significantly lower in the NPs‐Ce6 + PCC + US group, confirming that hypoxia relief inhibited SLC7A11 expression, thereby reducing Cys transport and GSH synthesis in cells. Additionally, malondialdehyde (MDA), a key marker of lipid peroxidation (LPO), was quantified to assess LPO levels (Figure 6f). C11‐BODIPY^581/591^, a probe specific to LPO, showed marked red fluorescence decay and green fluorescence intensification in the NPs‐Ce6 + PCC + US group, indicating increased LPO due to oxygenation‐enhanced ROS generation (Figure 6g). To validate the role of ferroptosis in cancer cell killing, inhibitors like deferoxamine (DFO) and ferrostatin‐1 (Fer‐1) were added to the NPs‐Ce6 + PCC + US group, resulting in a concentration‐dependent increase in cell viability (Figure 6h; Figure S20, Supporting Information). Moreover, Fer‐1 and DFO did not induce significant toxicity to the cells, thereby excluding their potential cytotoxic effects (Figure S21, Supporting Information). NPs‐Ce6 + PCC + US effectively elevated O_2_ levels to alleviate the hypoxic environment and led to decreased SLC7A11 expression, reduced GSH biosynthesis, diminished GPX4 activity, increased intracellular LPO, and ultimately, inducing ferroptosis. In summary, hypoxia induced the expression of HIF‐1α, which consequently increased SLC7A11 protein expression. SLC7A11, a specific amino acid transporter, is crucial in regulating ferroptosis. Reducing hypoxia could downregulate SLC7A11 expression, thus impeding Cys transport into cells. This reduction led to lower intracellular Cys levels and decreased GSH biosynthesis, which indirectly inhibited GPX4 activity, resulting in LPO accumulation and ultimately inducing ferroptosis. Furthermore, SDT‐mediated ^1^O_2_ generation disrupted intracellular ferritin, releasing ferric iron and further depleting GSH (Figure 6i). Based on the aforementioned data and analysis, it can be concluded that mitigating tumor hypoxia not only bolsters the therapeutic efficacy of SDT but also augments ferroptosis.

**Figure 7:**
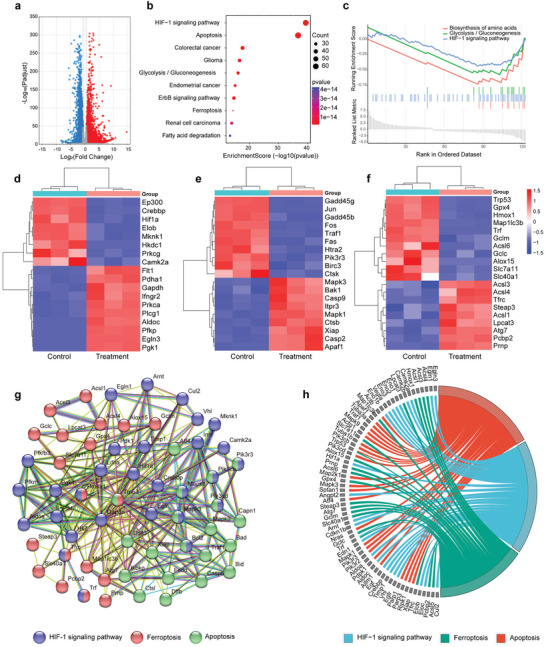
Underlying therapeutic mechanism. a) Volcano plot of identified DEGs between the control group and NPs‐Ce6 + PCC + US group, in which red dots represent upregulated genes and blue dots represent downregulated genes with a fold change ≥ 1.5 (or ≤ −1.5), *p* < 0.05. b) KEGG pathway enrichment analysis of identified DEGs. c) GSEA analysis of identified DEGs. Heat map of differential genes associated with d) HIF‐1α, e) apoptosis, and f) ferroptosis in 4T1 cells after different treatments. g) Protein–protein interaction network of differential genes associated with HIF‐1α, ferroptosis, and apoptosis. h) KEGG enrichment chord diagram of identified DEGs. NPs‐Ce6 concentration: 400 µg mL^−1^, PCC concentration: 5 × 10^7^ cell mL^−1^.

After confirming the anti‐cancer effect in vitro, RNA sequencing was conducted to further elucidate the potential therapeutic mechanism. As shown in **Figure** **7**a, there were 56981 differentially expressed genes (DEGs), with 46328 upregulated (red dots) and 10653 downregulated genes (blue dots) in the volcano. These DEGs underwent Kyoto Encyclopedia of Genes and Genomes (KEGG) pathway analysis for deeper insights into NPs‐Ce6 + PCC + US on 4T1 tumor cell therapy. This treatment led to significant gene enrichment changes related to the HIF‐1α signaling pathway, apoptosis, ferroptosis, GSH metabolism, and glycolysis (Figure 7b). Additionally, gene set enrichment analysis (GSEA) of the DEGs, using the Molecular Signature Database (MSigDB), revealed enrichment in pathways associated with disrupted amino acid biosynthesis and the HIF‐1α signaling pathway, suggesting that the treatment resulted in tumor cell death and hypoxia remission, potentially via photosynthesis enhanced by sonoafterglow (Figure 7c). To further explore this mechanism, heat maps of genes related to the HIF‐1α signaling pathway, apoptosis, and ferroptosis were generated (Figure 7d–f), confirming the activation of these pathways. Moreover, protein‐protein interaction network analysis of differentially expressed genes linked to the HIF‐1α signaling pathway, apoptosis, and ferroptosis were conducted (Figure 7g). Downregulation of HIF‐1α and GPX4, as observed in the differential expression analysis, contributed to hypoxia relief and ferroptosis induction (Figures S22 and S23, Supporting Information). Last, crossover analysis based on KEGG highlighted gene function enrichment (Figure 7h), confirming changes in the HIF‐1α signaling pathway, apoptosis, and ferroptosis, which suggested a vital role of NPs‐Ce6 + PCC + US in tumor therapy.

### 2.6 In Vivo Antitumor Performance

**Figure 8:**
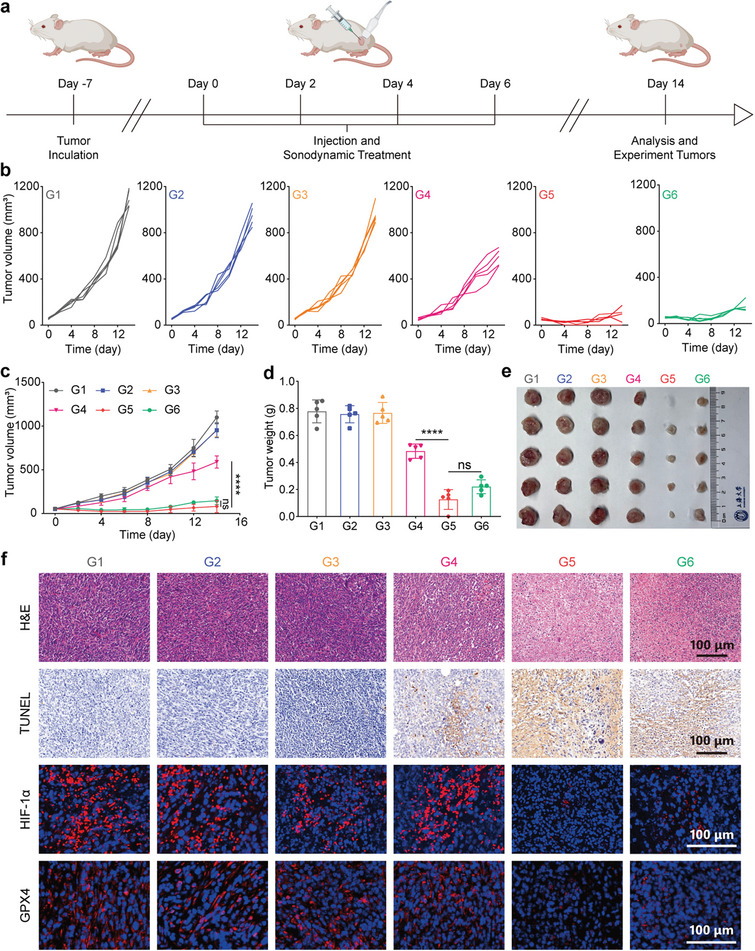
In vivo evaluation for antitumor therapy. a) Schematic illustration showing the process of sonosynthesis‐mediated SDT (Created with BioRender.com). b) Individual tumor growth of tumor‐bearing mouse in each group G1) PBS control; G2) US only; G3) NPs‐Ce6 + PCC; G4) NPs‐Ce6 + US; G5) NPs‐Ce6 + PCC + US and G6) NPs‐Ce6 + PCC + US 3 mm. c) Tumor growth curve of each group (*n* = 5). d) Weight and e) digital photo of harvested tumors from the 4T1 tumor‐bearing mice after different treatments (*n* = 5). f) H&E staining, TUNEL staining, HIF‐1α immunohistochemical staining, and GPX4 immunohistochemical staining images of 4T1‐tumor sections from the tumor‐bearing mice different treatments. (NPs‐Ce6 concentration: 500 µg mL^−1^, PCC concentration: 1 × 10^8^ cell mL^−1^, 1.5 W cm^−2^, 5 min) Data are presented as mean values ± SD. Statistical differences of *p* values were determined by One‐way ANOVA. ^****^ *p* < 0.0001.

Based on the substantial induction of ferroptosis and the amelioration of hypoxia achieved in vitro with NPs‐Ce6 + PCC‐mediated SDT (500 µg mL^−1^ NPs‐Ce6 and 1 × 10^8^ cells mL^−1^ PCC, 100 µL), we investigated the antitumor effect in vivo. 4T1 tumor‐bearing mice were randomly divided into six groups: G1) PBS control; G2) US only; G3) NPs‐Ce6 + PCC (simultaneously injecting NPs‐Ce6 and PCC; i.t. injection of the mixture of NPs‐Ce6 (500 µg mL^−1^) and PCC (1 × 10^8^ cell mL^−1^), 100 µL); G4) NPs‐Ce6 + US; G5) NPs‐Ce6 + PCC + US and G6) NPs‐Ce6 + PCC + US 3 mm (coating with 3 mm chicken breast to mimic deep tumor therapy) (**Figure** **8**a). Notably, Group G2 (US) and Group G3 (NPs‐Ce6 + PCC) exhibited a rapid tumor growth, similar to the Group G1 (control). In contrast, Group G4 (NPs‐Ce6 + US) showed a significant reduction in tumor progression, with a 41.9% inhibition rate (Figure S24, Supporting Information). Remarkably, Group G5 (NPs‐Ce6 + PCC + US) and Group G6 (NPs‐Ce6 + PCC + US, 3 mm) demonstrated significantly enhanced tumor suppression, with suppression rates of 92.2% and 86.0%, respectively, which was attributed to ferroptosis and hypoxia alleviation‐improved SDT (Figure 8b,c; Figure S25, Supporting Information). The efficacy in Group G6 highlights the high potential of this therapeutic strategy for treating deep‐seated tumors based on the high tissue‐penetrating capability of the US. Ex vivo analysis of representative tumors confirmed this, with tumor weights measured at the end of therapy (Figure 8d,e). Biosafety assessments indicated no significant body weight changes (Figure S26, Supporting Information) in all experimental groups during the treatment period. Furthermore, comprehensive assessments of hematology parameters and blood biochemical indexes were conducted (Figure S27, Supporting Information). The results demonstrate no significant differences across the different groups, indicating remarkable biocompatibility without noticeable hepatic and renal toxicity. Additionally, the histopathological examinations through H&E staining of the major organs such as the heart, liver, spleen, lung, and kidney suggest neglectable toxicity associated with the treatment (Figure S28, Supporting Information).

The antitumor effects were further corroborated by hematoxylin and eosin (H&E), Ki‐67, TdT‐mediated dUTP‐biotin nick end labeling (TUNEL), and cleaved caspase‐3 staining of tumor sections. These analyses revealed the highest levels of apoptosis and necrosis, coupled with the lowest proliferation, in the tumor tissue from Group G5 (Figure 8; Figure S25, Supporting Information). Considering hypoxia typically triggers HIF‐1α, which transactivates various signaling molecules including vascular endothelial growth factor (VEGF) and CD31 to promote angiogenesis and tumor growth, immunohistochemical staining was used to assess HIF‐1α, VEGF, and CD31 levels. Notably, Groups G5 and G6 exhibited significantly reduced expression of HIF‐1α, CD31, and VEGF, highlighting the reduction of hypoxia in the tumor region (Figure 8f; Figures S29,  S30, Supporting Information). Given that GPX4 reduction is a hallmark event in the ferroptosis process, we conducted a thorough investigation of GPX4 expression in tumor tissues post‐therapy. Immunohistochemical analysis displayed a significant decrease of GPX4 expression in Groups G5 and G6 compared to the other groups, further supporting the occurrence of ferroptosis within the tumors. These results validated the function of the sonosynthesis‐based SDT platform in effectively remodeling the tumor hypoxic microenvironment, which could not only enhance the therapeutic efficacy of SDT but also trigger ferroptosis in cancer cells.

## 3 Conclusion

In summary, this work has provided a proof‐of‐concept on sonosynthesis and established a therapeutic strategy that augments SDT by sonosynthetic cyanobacteria oxygenation. The engineered NPs‐Ce6, featuring inherent sonosensitizer and sonoafterglow properties, enables the generation of ^1^O_2_ and activation of cyanobacteria for US‐activated photosynthetic oxygenation, thus augmenting the therapeutic effects and outcomes of SDT. Stimulating cyanobacterial photosynthesis via US‐activated sonoafterglow overcomes the limitations of external light penetration and avoids potential side effects of prolonged light exposure. Our findings, both in vivo and in vitro, confirm the capability of sonoafterglow to trigger photosynthetic oxygenation in cyanobacteria, effectively remodeling the tumor hypoxic microenvironment. This desirable effect not only enhances the therapeutic efficacy of SDT but also triggers ferroptosis in cancer cells. The developed sonosynthesis‐based therapeutic platform effectively addresses the twin challenges of hypoxia and light penetration by ultrasound‐photo conversion, heralding a new era in aerobic precision medicine, particularly for in vivo applications.

## Conflict of Interest

The authors declare no conflict of interest.

## Supporting information

Supporting Information

## Data Availability

The data that support the findings of this study are available from the corresponding author upon reasonable request.
