# Supplementary material for: Sonosynthetic Cyanobacteria Oxygenation for Self‐Enhanced Tumor‐Specific Treatment
Source: Adv Sci (Weinh). 2024 Jun 12;11(29):2400251. doi: 10.1002/advs.202400251 (PMC11304326; doi:10.1002/advs.202400251)
Supplement: Supplementary file 1 — Supporting Information [file ADVS-11-2400251-s001.pdf]

ADVANCED SCIENCE
Open Access

Supporting Information

for *Adv. Sci.*, DOI 10.1002/advs.202400251

Sonosynthetic Cyanobacteria Oxygenation for Self-Enhanced Tumor-Specific Treatment

Zhenyu Yang, Xiu Shen, Junyi Jin, Xiaoyan Jiang, Wenqi Pan, Chenyao Wu, Dehong Yu, Ping Li, Wei Feng* and Yu Chen**

Supporting Information
©Wiley-VCH 2023
69451 Weinheim, Germany

Sonosynthetic Cyanobacteria Oxygenation for Self-enhanced Tumor-Specific Treatment

Zhenyu Yang, Xiu Shen, Junyi Jin, Xiaoyan Jiang, Wenqi Pan, Chenyao Wu, Dehong Yu, Ping Li,* Wei Feng,* Yu Chen*

Abstract: Photosynthesis, essential for life on earth, sustains diverse processes by providing nutrition in plants and microorganisms. Especially, photosynthesis has been increasingly applied in disease treatments, but its efficacy is substantially limited by the well-known low penetration depth of external light. Here, we report ultrasound-mediated photosynthesis for enhanced sonodynamic tumor therapy using organic sonoafterglow (ultrasound-induced afterglow) nanoparticles combined with cyanobacteria, demonstrating the proof-of-concept sonosynthesis (sonoafterglow-induced photosynthesis) in cancer therapy. Chlorin e6, a typical small-molecule chlorine, is formulated into nanoparticles to stimulate cyanobacteria for sonosynthesis, which serves three roles, *i.e.*, overcoming the tissue-penetration limitations of external light sources, reducing hypoxia, and acting as a sonosensitizer for in vivo tumor suppression. Furthermore, sonosynthetic oxygenation suppresses the expression of hypoxia-inducible factor 1α, leading to reduced stability of downstream SLC7A11 mRNA, which results in glutathione depletion and inactivation of glutathione peroxidase 4, thereby inducing ferroptosis of cancer cells. This study not only broadens the scope of microbial nanomedicine but also offers a distinct direction for sonosynthesis.

DOI: 10.1002/anie.2023XXXX

Table of Contents

Experimental Procedures **3**
Materials and reagents 3
Cyanobacteria and BG11 culture medium 3
Synthesis of NPs-Ce6 3
Apparatus and characterization 3
In vitro singlet oxygen generation detection 3
ESR measurements 4
Cell culture 4
In vitro cytotoxicity assay 4
Cellular uptake 4
Cell apoptosis analysis 4
In vitro iron assessment 4
Detection of intracellular 1O2 production 4
In vitro cellular oxygenation detection 5
Western blot analysis 5
Animal models 5
In vivo antitumor efficacy 5
Statistical analysis 5
Results and Discussion **6**
Figure S1 6
Figure S2. 6
Figure S3. 7
Figure S4 7
Figure S5 8
Figure S6 9
Figure S7 10
Figure S8 11
Figure S9 11
Figure S10 12
Figure S11 12
Figure S12 13
Figure S13 13
Figure S14 14
Figure S15 15
Figure S16 16
Figure S17 16
Figure S18 17
Figure S19 17
Figure S20 18
Figure S21 18
Figure S22 20
Figure S23 20
Author contributions **21**

Experimental Procedures

Materials and reagents

Chlorin e6 (Ce6), PEG-b-PPG-b-PEG (F127, average Mn ~12000), 1,3-diphenylisobenzofuran (DPBF), 9,10-anthracenediyl-bis (methylene) dimalonic acid (ABDA), and 2,2,6,6-tetramethylpiperidine (TEMP) were purchased from Shanghai Macklin Biochemical Co. Ltd. Cell counting kit-8 (CCK-8), 2′,7′-dichlorodihydrofluorescein diacetate (DCFH-DA), calcein acetoxymethyl ester (Calcein AM)/propidium iodide (PI), C11-BODIPY581/591, annexin V-fluorescein isothiocyanate (FITC)/PI co-staining kit, glutathione (GSH) assay kit, cysteine (Cys) assay kit, malondialdehyde (MDA) assay kit, mitochondrial membrane potential assay kit with JC-1, ferrostatin-1 (Fer-1), paraformaldehyde (4%), and triton X-100 (0.1%) were purchased from Beyotime Biotechnology. (Shanghai, China). Roswell Park Memorial Institute (RPMI) 1640 medium, Dulbecco’s Modified Eagle medium (DMEM), phosphate buffer solution (PBS), fetal bovine serum (FBS), streptomycin, and penicillin were purchased from Invitrogen Gibco (Carlsbad, CA). C11-BODIPY (581/591) dye, anti-GPX4 antibody (Catalog No. A13309), anti-SLC7A11 antibody (Catalog No. A2413), anti-HIF-1α antibody (Catalog No. A22041) and HIF-1α ELISA assay kit were purchased from Abclonal Technology Co. Ltd. (Wuhan, China). Ultrapure water (Unique-R10, 18.2 MΩ) was used through the experiment.

Cyanobacteria and BG11 culture medium

Cyanobacteria (*Synechococcus* 7942) were obtained from the Institute of Plant Physiology and Ecology (Shanghai, China). The stock solutions were prepared as follows: Stock 1: $C_6H_8O_7 \cdot H_2O$ (6.567 g L^{-1}), $C_6H_{11}FeNO_7$ (6 g L^{-1}), $EDTANa_2 \cdot 2H_2O$ (1.107 g L^{-1}). Stock 2: H_3BO_3 (2.86 g L^{-1}), $MnSO_4 \cdot H_2O$ (1.545 g L^{-1}), $ZnSO_4 \cdot 7H_2O$ (0.222 g L^{-1}), $CuSO_4 \cdot 5H_2O$ (0.079 g L^{-1}), Na2MoO4·$2H_2O$ (0.391 g L^{-1}), $CoCl_2 \cdot 6H_2O$ (0.0404 g L^{-1}). Stock 3: $K_2HPO_4 \cdot 3H_2O$ (52 g L^{-1}), Na_2CO_3 (20 g L^{-1}). Then, cyanobacteria were cultured in a sterile 1 × BG11 medium containing $NaNO_3$ (1.5 g), $MgSO_4 \cdot 7H_2O$ (0.075 g), $CaCl_2 \cdot 2H_2O$ (0.036 g), HEPES (4.76 g), Stock 1 (1 mL), Stock 2 (1 mL), and Stock 3 (1 mL) at 25 ℃ under light with oscillation process.

Synthesis of NPs-Ce6

A bottom-up approach (nanoprecipitation) is used to synthesize the NPs-Ce6 from the homogeneously dissolved tetrahydrofuran (THF) solution of Ce6. Briefly, PEG-b-PPG-b-PEG (20 mg mL^{-1}) and Ce6 (0.15 mg mL^{-1}) were dissolved into THF solution (5 mL). The mixture was then injected into ultrapure water (45 mL) under magnetic stirring for 30 min. Then THF was evaporated with a rotary evaporator. Finally, the resulting solution of NPs-Ce6 was received by ultracentrifugation with 30 K centrifugal filter units (Millipore) and the concentrated solution of NPs-Ce6 was diluted with 1 × PBS buffer. The obtained nanoparticle dispersion was stored in the dark at 4 °C.

Apparatus and characterization

Transmission electron microscopy (TEM) was tested by JEM-F200 (JEOL, Japan). Scanning electron microscopy (SEM) was tested by JSM-6700F (JEOL, Japan). DLS and Zeta potential were tested by nanosonosensitizer size analyzer (Zetasizer 3000HS, Malvern Instruments, UN). Ultraviolet-visible (UV–vis) absorbance spectra were performed on a UV-1800 UV-vis scanning spectrometer. Fluorescence spectra measurement was carried out on a F-7000 spectrofluorimeter. ROS were identified by an EMX plus-6/1 ESR spectrometer. The confocal laser scanning microscopy (CLSM) images were obtained by a CLSM 710 microscope. The sonoafterglow images were captured on a VISQUE In-Vivo Smart-LF imaging system. Flow cytometry was performed by using a Becton–Dickinson spectrophotometer. The cell viability was measured by SpectraMax iD5 microplate reader.

In vitro singlet oxygen generation detection

DPBF was utilized to study the singlet oxygen levels produced by different formulations for different irradiation time. DPBF (1 mg mL^{-1} in DMF, 25 μL) was mixed with 2 mL ultrapure water, NPs-Ce6, cyanobacteria and NPs-Ce6 with cyanobacteria (NPs-Ce6 concentration: 500 μg mL^{-1}, cyanobacteria concentration: 1 × 10^6 cell mL^{-1}). All samples were under ultrasound (US) irradiation for 0, 1, 2, 3 and 4 min (1 MHz, 1.5 W cm^{-2}, 50% duty cycle) and the absorption spectra of every minute were recorded using a UV-vis spectrophotometer.

ESR measurements

TEMP, a free-radical spin-trapping agent, was applied to detect the $^1O_2$ generation. ESR spectra were obtained by mixing 100 μL of NPs-Ce6 dispersion (500 μg mL^{-1}) and 100 μL of cyanobacteria (1 × 10^6 cell mL^{-1}) with 1 μL of TEMP (10 mM) under US irradiation for 2 min (1 MHz, 1.5 W cm^{-2}, 50% duty cycle).

Cell culture

4T1 murine breast cancer cell lines and 3T3 murine embryonic fibroblast cell lines were purchased from the cell bank of Chinese Academy of Sciences (CBCAS, Shanghai). 4T1 cancer cells were cultured in RPMI 1640 medium supplemented with 10% fetal bovine serum and 1% penicillin/streptomycin at 37 ℃ in a humidified atmosphere with 5% CO_2.

In vitro cytotoxicity assay

4T1 cells/3T3 cells were seeded into 96-well plates (cell density = 10^4 cells per disk) and cultured for 24 h. Then, the fresh culture medium containing NPs-Ce6 (0, 100, 200, 300, 400, and 500 μg mL^{-1}) or cyanobacteria (0, 2.5, 5, 7.5, 10, and 12.5 × 10^7 cell mL^{-1}) was added into the plates for 24 and 48 h, respectively. Finally, the typical CCK-8 assay was conducted according to the kit instruction.

Cellular uptake

4T1 cells were seeded into confocal bottom-dishes (cell density = 10^5 cells per disk) and cultured. Then the cell culture medium was replaced with fresh culture medium containing NPs-Ce6 (400 μg mL^{-1}) for 0, 2, 4 and 8 h, respectively. The treated 4T1 cells were washed twice with PBS under CLSM observation.

Cell apoptosis analysis

The 4T1 cells were seeded at a density of 1×10^5 cells/well into 6-well plates and cultured with RPMI-1640 medium containing 10% FBS for 24 h. The cells were co-incubated with different treatment including for 12 h. For treatment groups, the cells were exposed to the US for sonication for 5 min (1 MHz, 1.2 W cm^{-2}, 50% duty cycle), and cultured for another 12 h. Then trypsinised, washed with medium and centrifuged at 2000 rpm for 5 min. The cells were resuspended in 1 mL binding buffer containing Annexin V-FITC (10 μM) and PI (10 μM). The cells were incubated for 15 min in a dark environment. Lastly, the results were analyzed by flow cytometry.

In vitro iron assessment

The cytosolic iron level was measured by calcein acetoxymethyl ester (CAL-AM). The membrane-permeant CAL-AM could be loaded into cells swiftly and cleaved to the fluorescent substance CAL, which can be quenched by permeant iron (+3 or +2) chelators. 4T1 cells were incubated fresh culture medium containing NPs-Ce6 and NPs-Ce6 + cyanobacteria (NPs-Ce6: 400 μg mL^{-1}, cyanobacteria: 5 × 10^7 cell mL^{-1}) in 24-well plates for 12 h. After the cells were irradiated with the sonication for 5 min (1 MHz, 1.2 W cm^{-2}, 50% duty cycle). Then, the treated cells were supplemented with CAL-AM (0.2 μM) for 15 min in serum-free medium. Afterward, the cells were harvested and resuspended in PBS (400 μL) for flow cytometry analysis. The mean fluorescence intensity of the different groups was inversely proportional to the labile iron content.

Detection of intracellular $^1O_2$ production

DCFH-DA was used to defect the ROS production. In detail, 4T1 cancer cells (2 × 10^5 cell per dish) were seeded into confocal dish for 12 h at 37 ℃. Afterwards, the above cells were cultured for 12 h. Then, 4T1 cells were treated with fresh culture medium containing NPs-Ce6 (400 μg mL^{-1}) and NPs-Ce6 (400 μg mL^{-1}) + PCC (5 × 10^7 cell mL^{-1}). After incubation for 4 h, the cells were incubated with DCFH-DA (20 μM) for 15 min and washed for 3 times with PBS in the dark. Next, 4T1 cells were received with or without US irradiation for 5 min (1 MHz, 1.2 W cm^{-2}, 50% duty cycle). The fluorescence imaging was analyzed through CLSM.

In vitro cellular oxygenation detection

The cellular oxygenation in vitro was achieved by the confocal observations using the hypoxia-sensitive fluorescence probe $Ru(dpp)_3Cl_2$. 4T1 cells were seeded onto the confocal disk at a cell density of 10^5 cells/disk. After allowing the cells to attach for 12 h, they were placed in a hermetically sealed culture bag with a commercial oxygen-depriving catalyst for another 12 h. Cells were then incubated with $Ru(dpp)_3Cl_2$ (10 μg mL^{-1} in full 1640 medium) in a hermetic environment for 3 h. After gently rinsing with cold PBS, the previous medium was replaced with 1 mL of full 1640 medium containing NPs-Ce6 and NPs-Ce6 + cyanobacteria (NPs-Ce6: 400 μg mL^{-1}, cyanobacteria: 5 × 10^7 cell mL^{-1}). 4T1 cells were exposed to US irradiation for 5 min (1 MHz, 1.2 W cm^{-2}, 50% duty cycle). After co-incubating of 2 h in the dark, the cells were observed under a confocal microscope (Ex: 488 nm, Em: 610 nm).

Western blot analysis

4T1 cells were seeded into 6-well plates and cultured for 24 h. Then, 4T1 cells were cultured with fresh culture medium containing NPs-Ce6 (400 μg mL^{-1}) and NPs-Ce6 (400 μg mL^{-1}) + PCC (5 × 10^7 cell mL^{-1}) for 4 h. Next, 4T1 cells were received with or without US irradiation for 5 min (1 MHz, 1.2 W cm^{-2}, 50% duty cycle), and cultured for another 6 h. Then 4T1 cells were washed with PBS and lysed to collect the protein. The PVDF membranes were blocked with 5% nonfat dry milk at room temperature for 1 h in the decoloring shaker and then incubated with anti–HIF-1α, anti-SLC7A11, anti-ferritin and anti-GPX4 overnight at 4 °C. The membranes were washed for three times in the decoloring shaker. Subsequently, the membranes were incubated with secondary antibodies for 30 min and washed for three times in the decoloring shaker subsequently. Enhanced chemiluminescence (ECL) reagent was then added and reacted for 1-2 min. The membranes were exposed in a darkroom and took images.

Animal models

All animal experiments were performed in strict compliance with the guidelines of the Institutional Animal Care and Use Committee of Shanghai University (Approval number: ECSHU 2022-053). Female BALB/c mice and female nude mice (6-8 weeks old) were purchased from Shanghai Legen Biotechnology Co., Ltd., and raised in Specific pathogen free class environment facility. The laboratory mice were housed under controlled conditions, namely 20-23 ℃ (± 0.5 ℃), 50-70% (± 10%) relative humidity, and a 12 h:12 h light:dark cycle. Water and food were available ad libitum for all animals.

In vivo antitumor efficacy

4T1 tumor-bearing mouse model was successfully established by subcutaneous injection of 1×10^7 tumor cells suspended in 150 μL RPMI-1640 medium into right hind leg of each BALB/c mice. All tumor-bearing were randomly divided into six groups (n = 5) once the tumor volume reached around 50-60 mm^3, and respectively treated with G1) PBS control; G2) US only; G3) NPs-Ce6 + PCC; G4) NPs-Ce6 + US; G5) NPs-Ce6 + PCC + US and G6) NPs-Ce6 + PCC + US 3mm (coating with 3 mm chicken breast to mimic deep tumor therapy). US irradiation (1.0 MHz, 1.5 W cm^{-2}, 50% duty cycle, 5 min). The tumor-bearing mice were intratumorally injected with different agents (PBS, NPs-Ce6 or NPs-Ce6 + PCC). After 0.5 h injection, group G2, G4, G5 and G6 were received US irradiation (1.0 MHz, 1.5 W cm^{-2}, 50% duty cycle, 5 min). The dose of NPs-Ce6 + PCC (100 μL) was 500 μg mL^{-1} of NPs-Ce6 and 1 × 10^8 cells mL^{-1} of PCC. Likewise, the dose of NPs-Ce6 (100 μL) was 500 μg mL^{-1}. Intratumoral injection was performed on day 0, day 2, and day 4. The whole experimental period was lasted for 2 weeks. The tumor volume and the mice weights were measured and calculated every 2 d to obtain the time variation curve. At the end of the observation period, all mice were sacrificed and dissected to obtain tumor tissue. The collected tumor tissues were weighed, photographed, and stained with hematoxylin and eosin (H&E), TdT-mediated dUTP-biotin nick end labeling (TUNEL), and Ki67 antibody.

Statistical analysis

All the data in the experiments were presented as mean ± SD and were repeated at least three times. Analysis of variance (ANOVA) was used for multiple comparisons, and two-tail Student's t-test was used for two-group comparisons. All statistical analyses were carried out using GraphPad Prism 8.0.2. The threshold for statistical significance was defined as $^*p < 0.05$, $^{**}p < 0.01$, $^{***}p < 0.001$ and $^{****}p < 0.0001$.

Results and Discussion

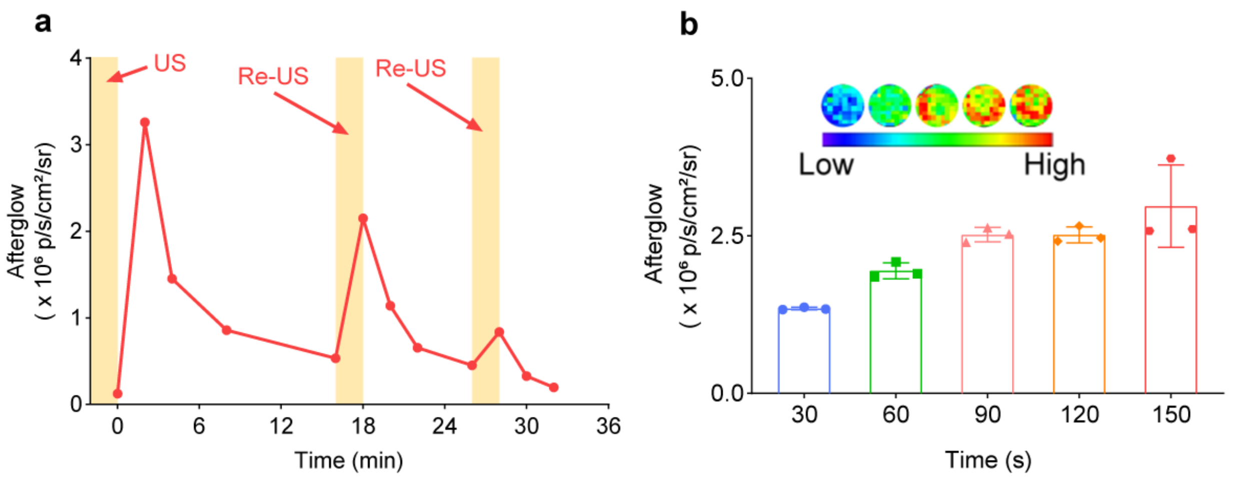

Figure S1. a) Sonoafterglow intensity of NPs-Ce6 after US irradiation for 2 min, and re-irradiation for 2 min. b) Time-dependent sonoafterglow intensity of NPs-Ce6 (n = 3). Data are presented as mean values ± SD.

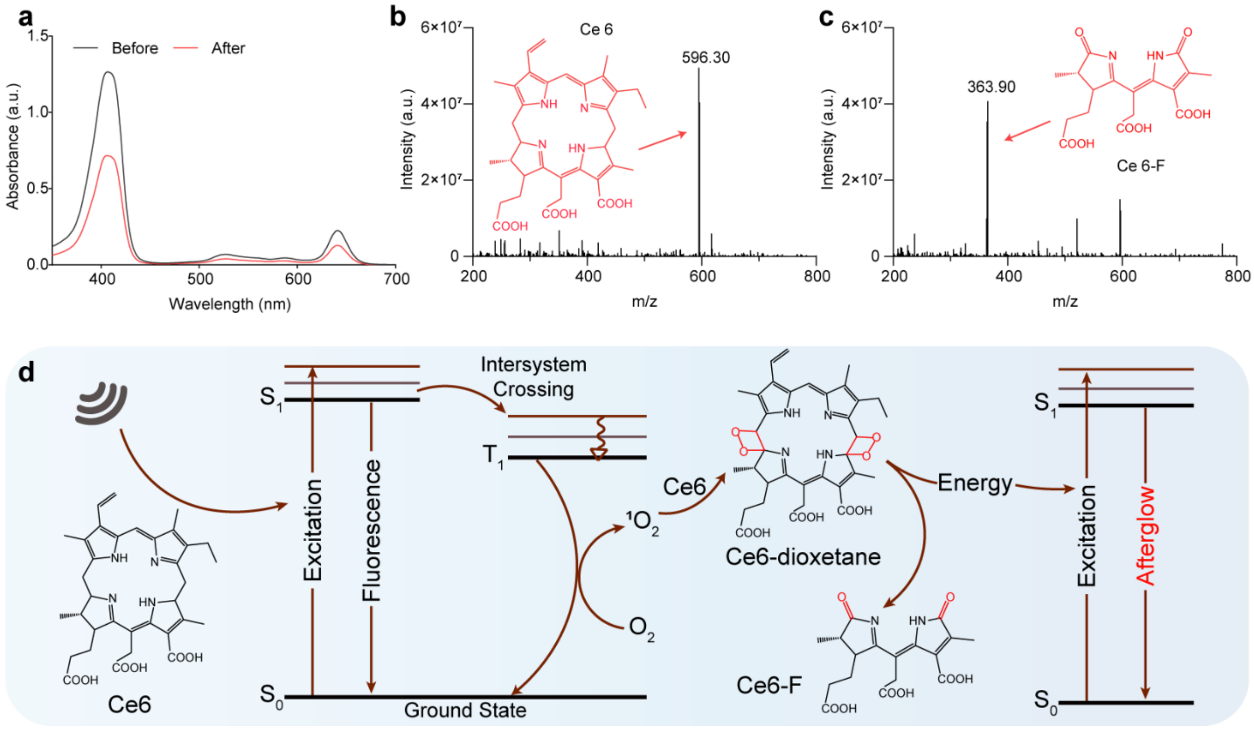

Figure S2. a) Ultraviolet-visible (UV-Vis) absorbance spectrum of NPs-Ce6 before and after US irradiation (NPs-Ce6 concentration: 500 μg mL^{-1}). b) MS analysis of Ce6 before US. Calculated for $C_{34}H_{36}N_4O_6$ [(M+H)]+: 596.26. Found: 596.30. c) MS analysis of Ce6 after US. Calculated for $C_{16}H_{16}N_2O_8$ [(M+H)]+: 364.09. Found: 363.90. d) Possible mechanism proposed for afterglow luminescence of Ce6 (S0, S1 and T1 represent singlet ground state, first excited singlet state and first excited triplet state, respectively).

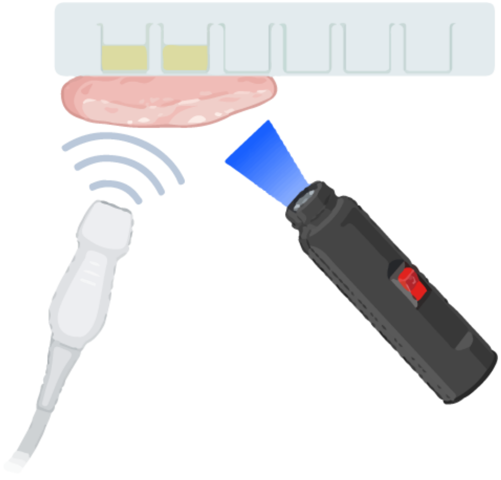

Figure S3. Schematic illustration showing the induction and detection of afterglow from NPs-Ce6 through chicken breast tissues following US or laser irradiation (Created with BioRender.com).

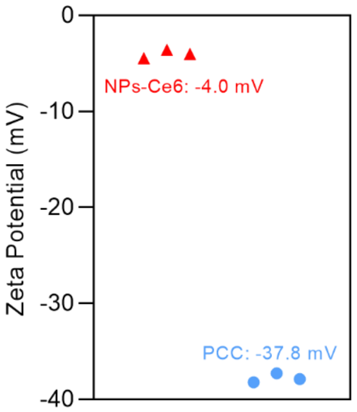

Figure S4. Zeta potentials of PCC and NPs-Ce6 (n = 3). Data are presented as mean values ± SD.

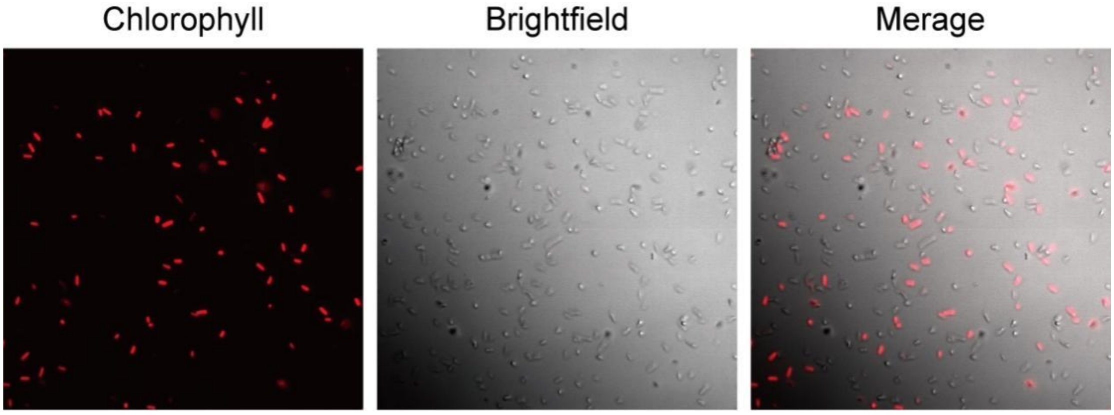

Figure S5. Fluorescence microscope image of PCC.

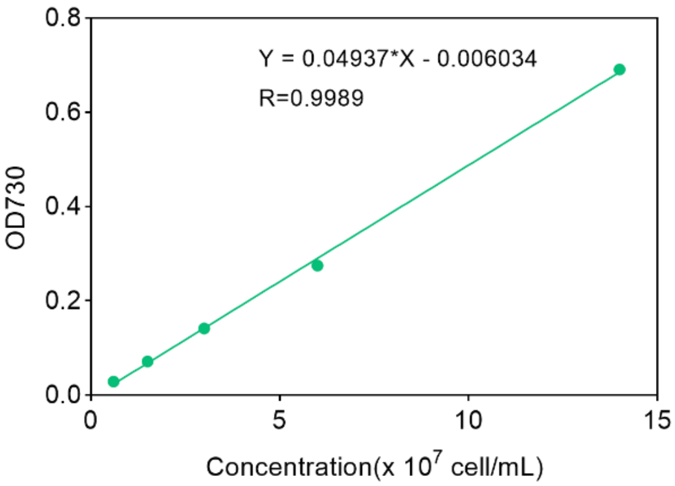

Figure S6. Standard curve of PCC at different concentrations.

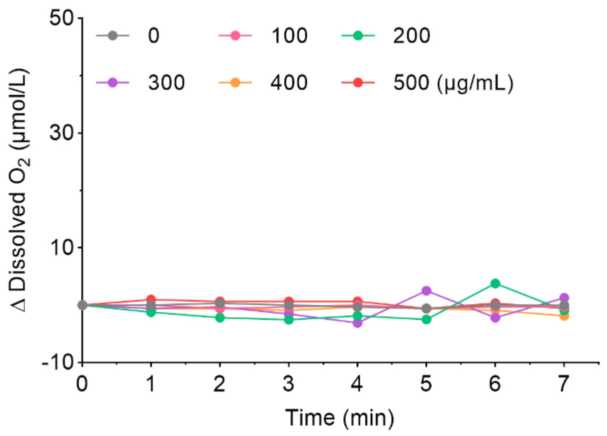

Figure S7. Concentration-dependent oxygenation of NPs-Ce6 under US irradiation.

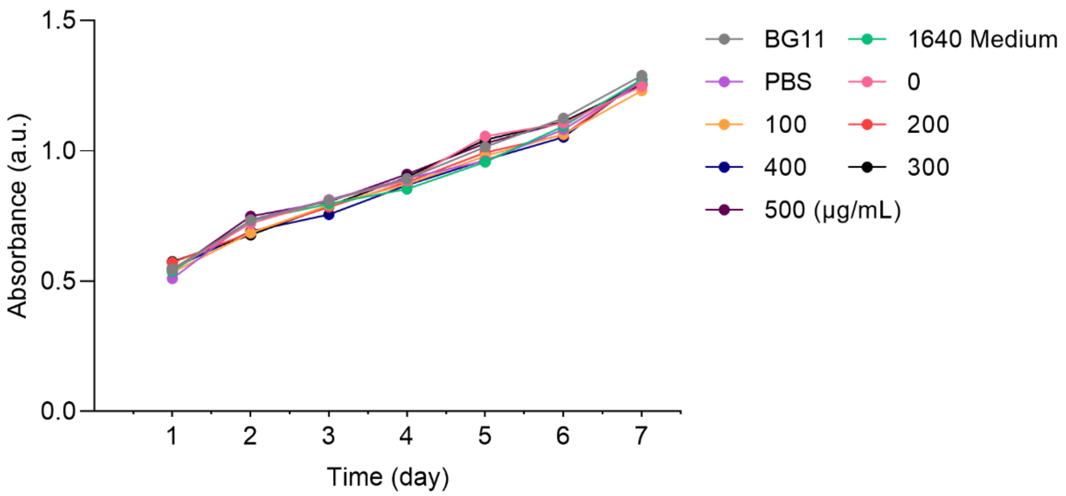

Figure S8. The growth curve of PCC in BG11 medium and 1640 medium or after treatment with different concentrations NPs-Ce6 in PBS (pH 6.4).

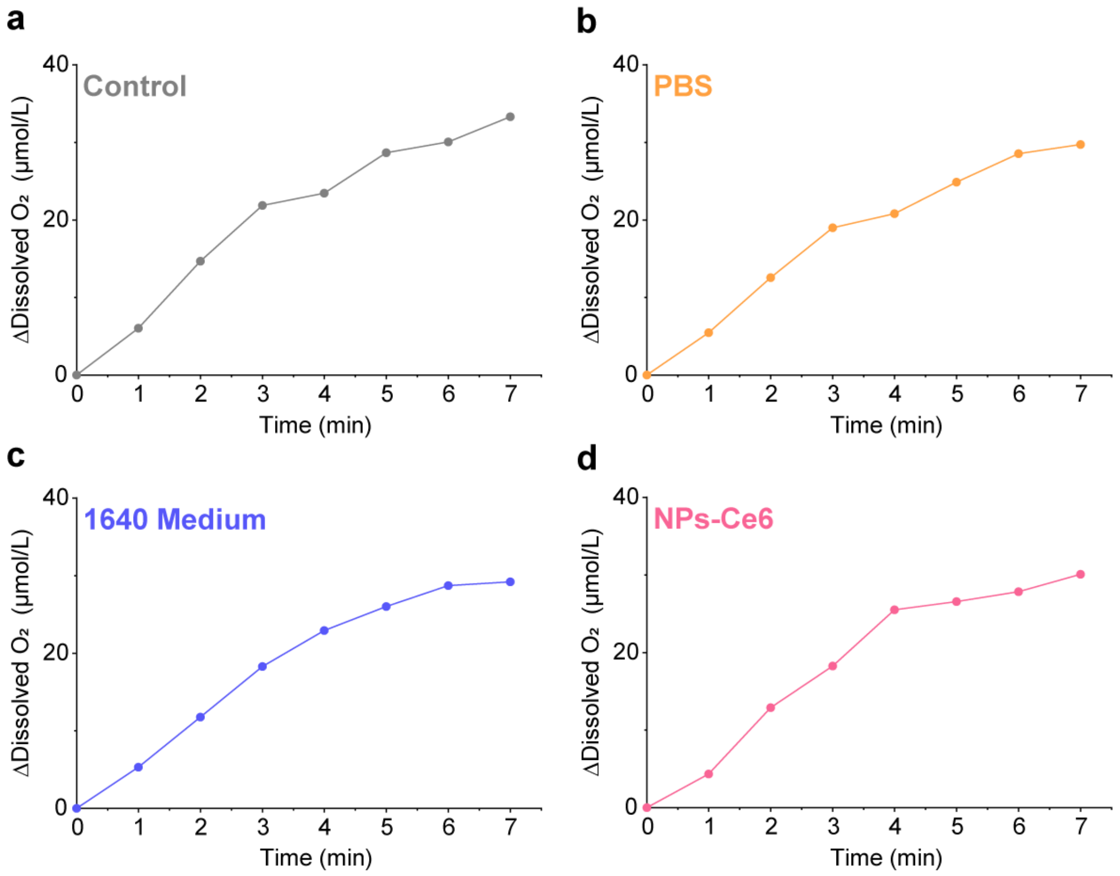

Figure S9. Dissolved oxygen levels of PCC (5×10^7 cell mL^{-1}) in a) BG11 medium, b) PBS (pH 6.4), c) 1640 medium and d) NPs-Ce6 (500 μg mL^{-1}) at day 7 under laser irradiation (660 nm, 1.5 W cm^{-2}). NPs-Ce6 concentration: 500 μg mL^{-1}, PCC concentration: 5×10^7 cell mL^{-1}.

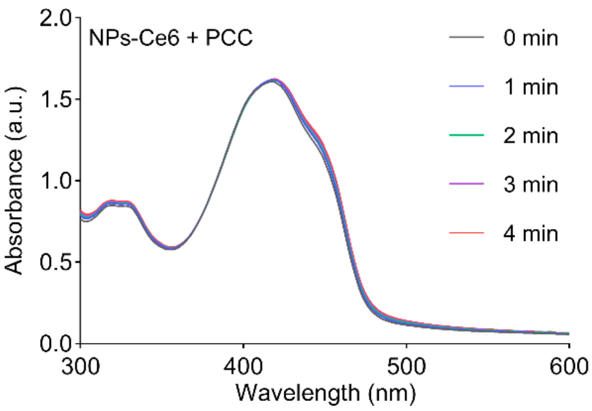

Figure S10. Time-dependent absorption changes of DPBF after treatment with NPs-Ce6 + PCC (NPs-Ce6 concentration: 500 μg mL^{-1}, cyanobacteria concentration: 1×10^6 cell mL^{-1}).

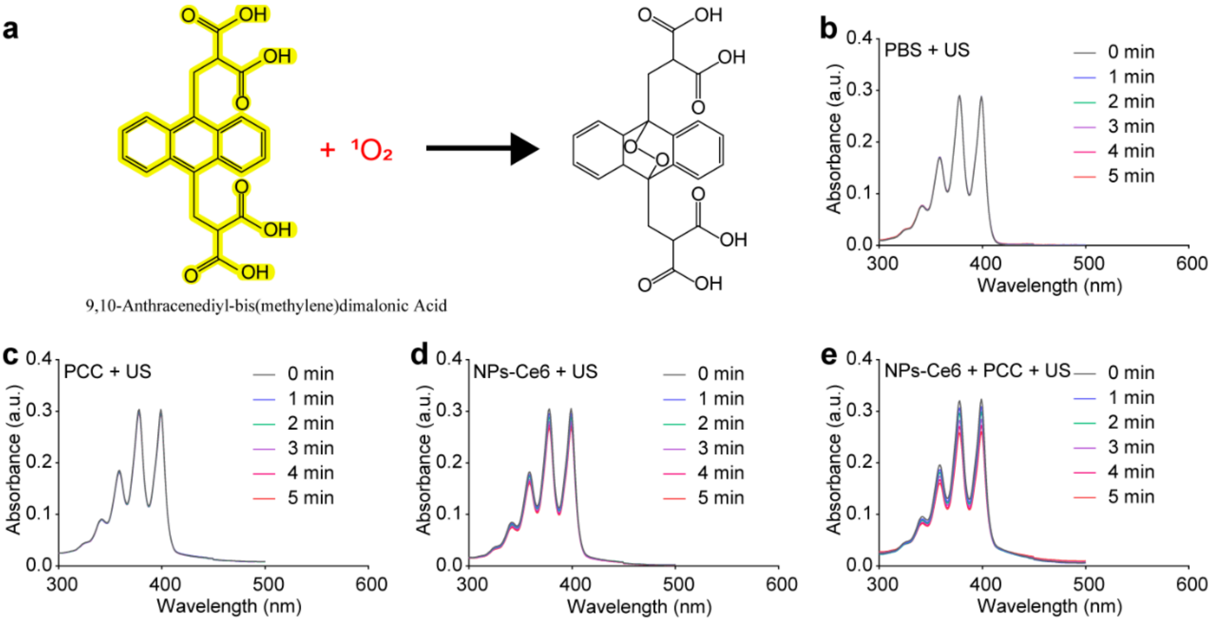

Figure S11. Sonodynamic performance tested by ABDA. a) ABDA structure changes before and after interacting with $^1O_2$. Time-dependent absorption changes of DPBF after treatment with b) PBS, c) PCC, d) NPs-Ce6 and e) NPs-Ce6 + PCC under US irradiation (NPs-Ce6 concentration: 500 μg mL^{-1}, PCC concentration: 1 × 10^6 cell mL^{-1}).

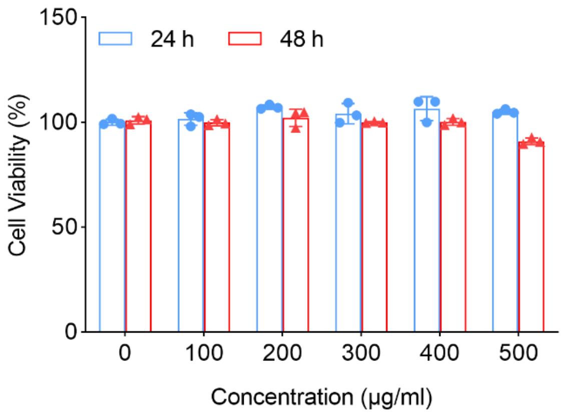

Figure S12. Relative viabilities of 4T1 cancer cells after treatment with NPs-Ce6 at different concentrations for 24 and 48 h (*n* = 3)**.** Data are presented as mean values ± SD.

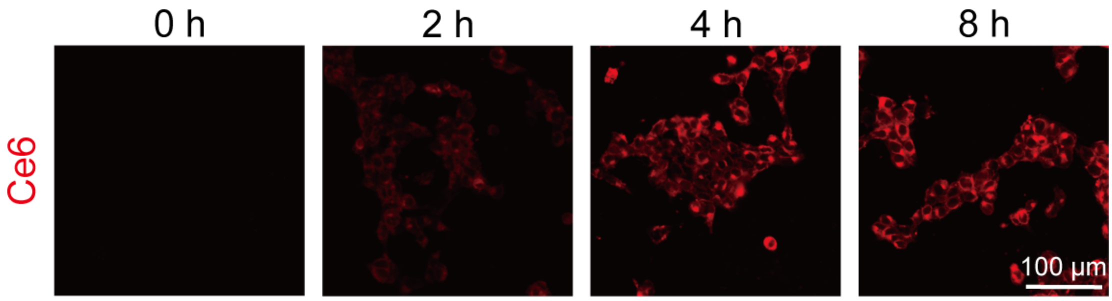

Figure S13. The uptake of 4T1 cancer cells after treatment with NPs-Ce6 at different timepoints (NPs-Ce6 concentration: 400 μg mL^{-1}).

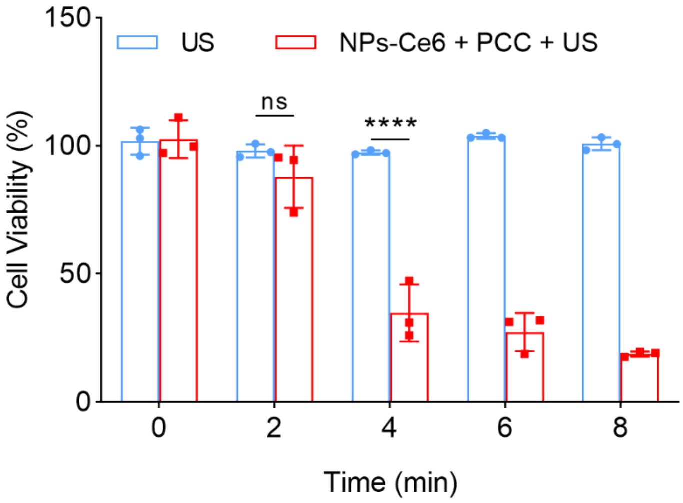

Figure S14. Relative viabilities of 4T1 cancer cells after different time of US irradiation (n = 3, NPs-Ce6 concentration: 400 μg mL^{-1}, PCC concentration: 5 × 10^7 cell mL^{-1}. Data are presented as mean values ± SD. Statistical differences of p values were determined by One-way ANOVA. ****$p < 0.0001$.

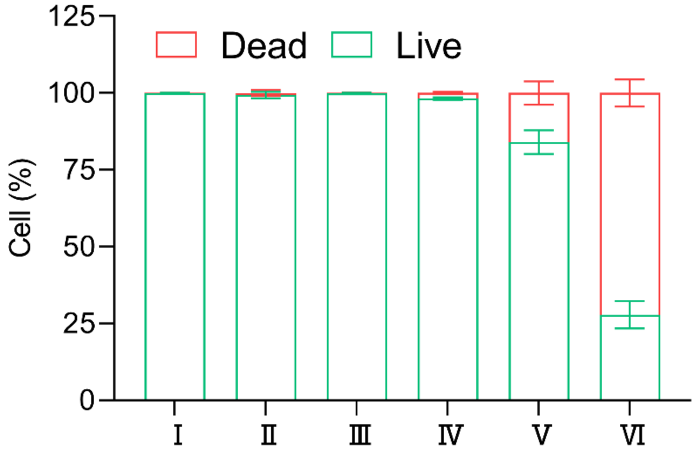

Figure S15. Fluorescence quantification of 4T1 cancer cells staining with Calcin-AM/PI after different treatments (n = 3, NPs-Ce6 concentration: 400 μg mL^{-1}, PCC concentration: 5 × 10^7 cell mL^{-1}). Data are presented as mean values ± SD. (I: Control; II: NPs-Ce6; III: NPs-Ce6 + PCC; IV: US; V: NPs-Ce6 + US; VI: NPs-Ce6 + PCC + US).

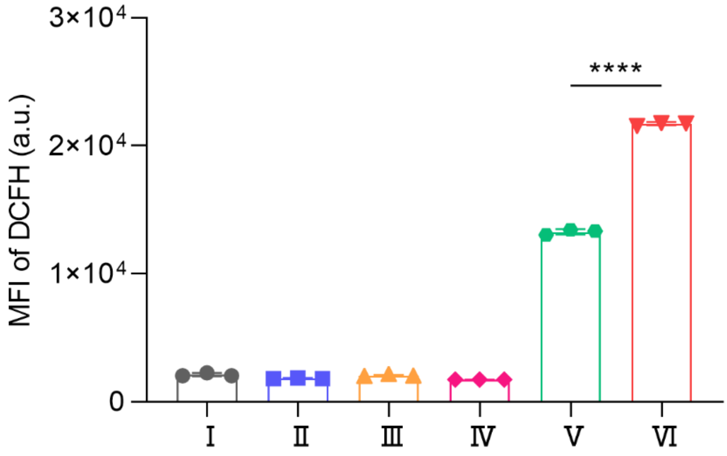

Figure S16. Mean fluorescence intensity of flow cytometry analysis of 4T1 cancer cells staining with DCFH-DA after indicated treatments (n = 3, NPs-Ce6 concentration: 400 μg mL^{-1}, PCC concentration: 5 × 10^7 cell mL^{-1}). Data are presented as mean values ± SD. Statistical differences of p values were determined by One-way ANOVA. ****$p < 0.0001$. (I: Control; II: NPs-Ce6; III: NPs-Ce6 + PCC; IV: US; V: NPs-Ce6 + US; VI: NPs-Ce6 + PCC + US).

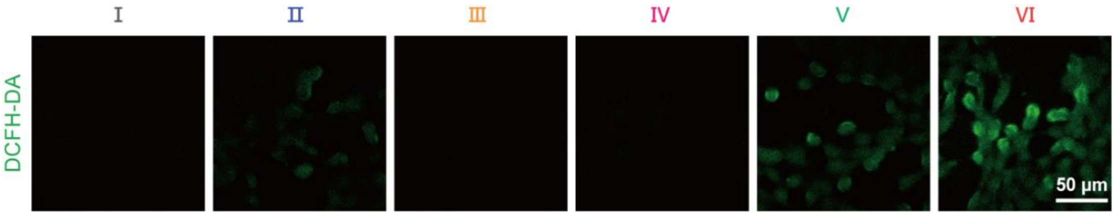

Figure S17. CLSM images of 4T1 cells staining with DCFH-DA probe after indicated treatments under hypoxic conditions. NPs-Ce6 concentration: 400 μg mL^{-1}, PCC concentration: 5 × 10^{7} cell mL^{-1}. (I: Control; II: NPs-Ce6; III: NPs-Ce6 + PCC; IV: US; V: NPs-Ce6 + US; VI: NPs-Ce6 + PCC + US).

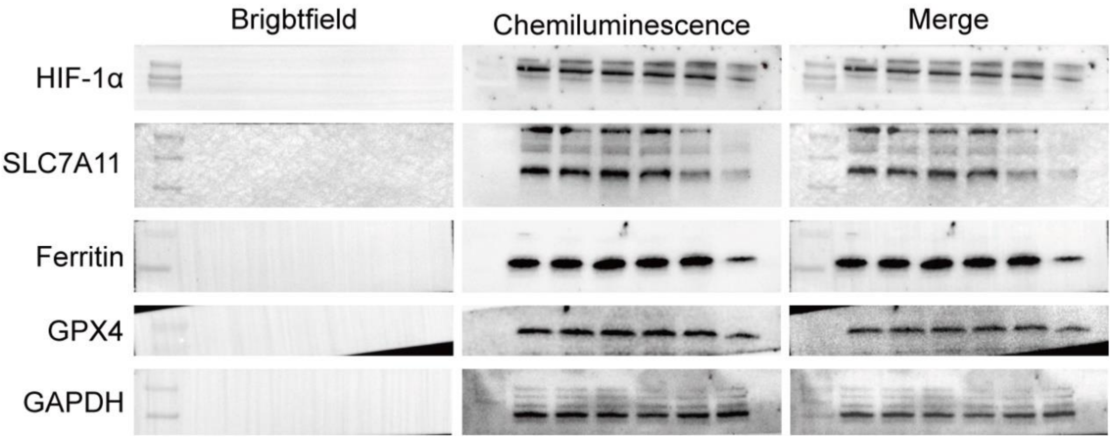

Figure S18. The raw western blot data of HIF-1α, SLC7A11, Ferritin, and GPX4 level in 4T1 cells treated after indicated treatments. NPs-Ce6 concentration: 400 μg mL^{-1}, PCC concentration: 5 × 10^{7} cell mL^{-1}.

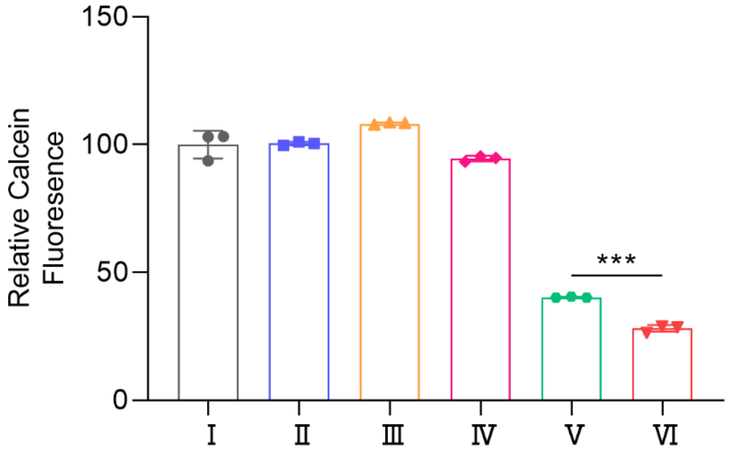

Figure S19. Flow cytometry assay of cytosolic iron levels in 4T1 cells after indicated treatments using calcein as an indicator (n = 3, NPs-Ce6 concentration: 400 μg mL^{-1}, PCC concentration: 5 × 10^{7} cell mL^{-1}). Data are presented as mean values ± SD. Statistical differences of p values were determined by One-way ANOVA. ***p < 0.001. (I: Control; II: NPs-Ce6; III: NPs-Ce6 + PCC; IV: US; V: NPs-Ce6 + US; VI: NPs-Ce6 + PCC + US).

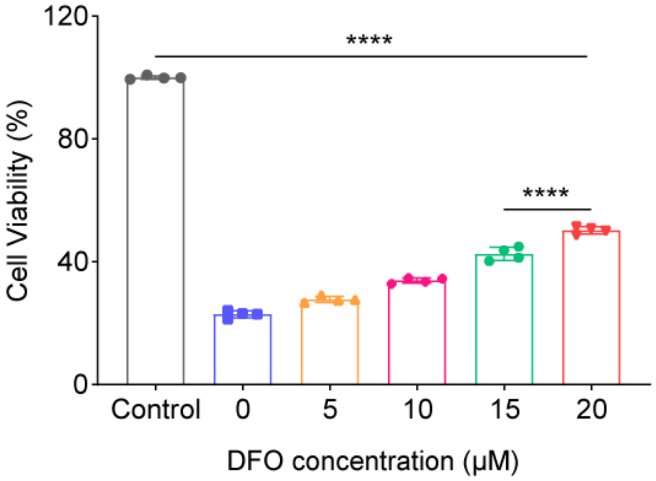

Figure S20. 4T1 cell viability after NPs-Ce6 + PCC + US in combination with the addition of different concentrations of DFO (n = 4, NPs-Ce6 concentration: 400 μg mL^{-1}, PCC concentration: 5 × 10^{7} cell mL^{-1}). Data are presented as mean values ± SD. Statistical differences of p values were determined by One-way ANOVA. ****p < 0.0001.

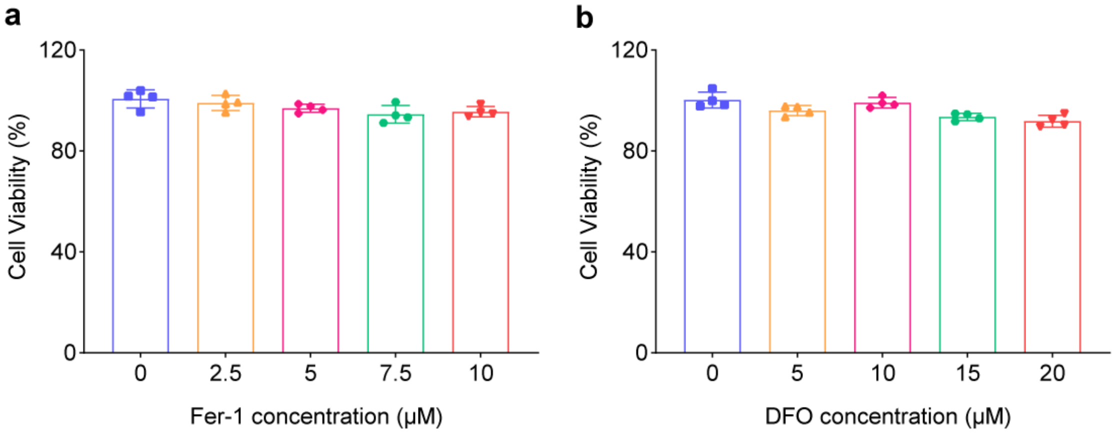

Figure S21. Relative viabilities of 4T1 cancer cells with a) Fer-1 and b) DFO at different concentrations ($n = 4$). Data are presented as mean values ± SD.

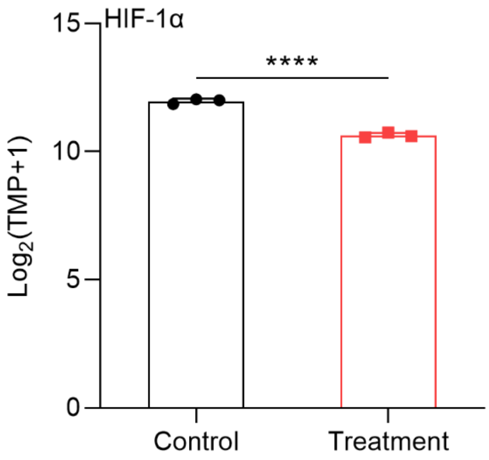

Figure S22. RNA sequencing to detect differential expression of HIF-1α after different treatments ($n = 3$, NPs-Ce6 concentration: 400 μg mL^{-1}, PCC concentration: 5×10^7 cell mL^{-1}.). Data are presented as mean values ± SD. Statistical differences of p values were determined by Student's t test. ****$p < 0.0001$.

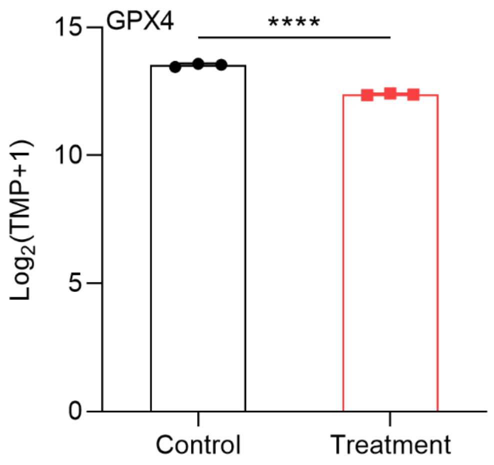

Figure S23. RNA sequencing to detect differential expression of GPX4 after different treatments (n = 3, NPs-Ce6 concentration: 400 μg mL^{-1}, PCC concentration: 5 × 10^{7} cell mL^{-1}.). Data are presented as mean values ± SD. Statistical differences of p values were determined by Student's t test. ****p < 0.0001.

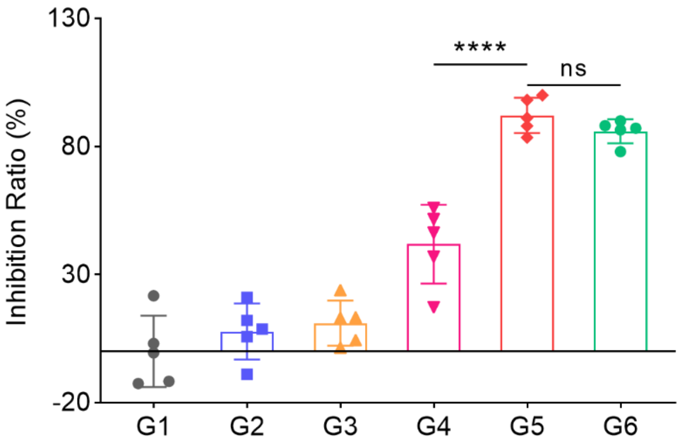

Figure S24. Tumor-growth inhibition rate after different treatments (n = 5, NPs-Ce6 concentration: 500 μg mL^{-1}, PCC concentration: 1 × 10^{8} cell mL^{-1}). Data are presented as mean values ± SD. Statistical differences of p values were determined by One-way ANOVA. ****p < 0.0001.

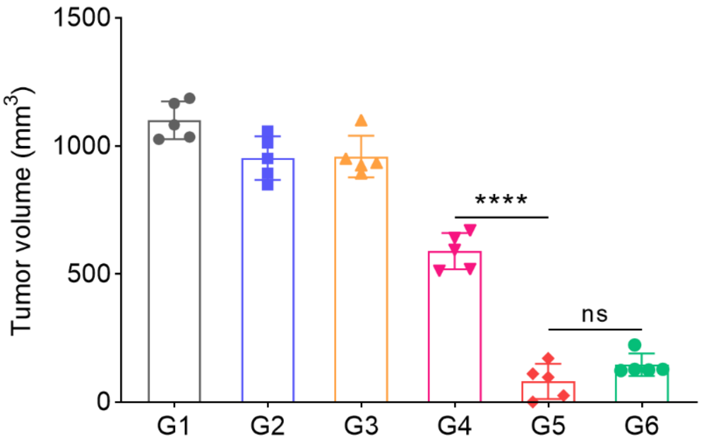

Figure S25. Tumor volumes of mice after different treatments on day 14 (n = 5, NPs-Ce6 concentration: 500 μg mL^{-1}, PCC concentration: 1 × 10^8 cell mL^{-1}). Data are presented as mean values ± SD. Statistical differences of p values were determined by One-way ANOVA. ****$p < 0.0001$.

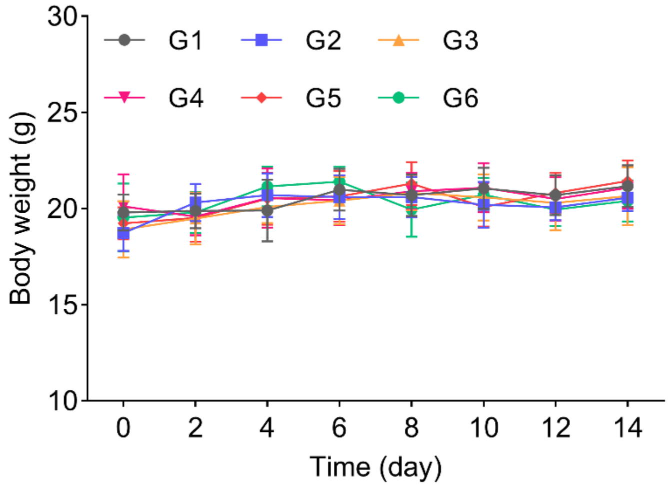

Figure S26. Average body weight changes of mice after different treatments (n = 5, NPs-Ce6 concentration: 500 μg mL^{-1}, PCC concentration: 1 × 10^8 cell mL^{-1}). Data are presented as mean values ± SD.

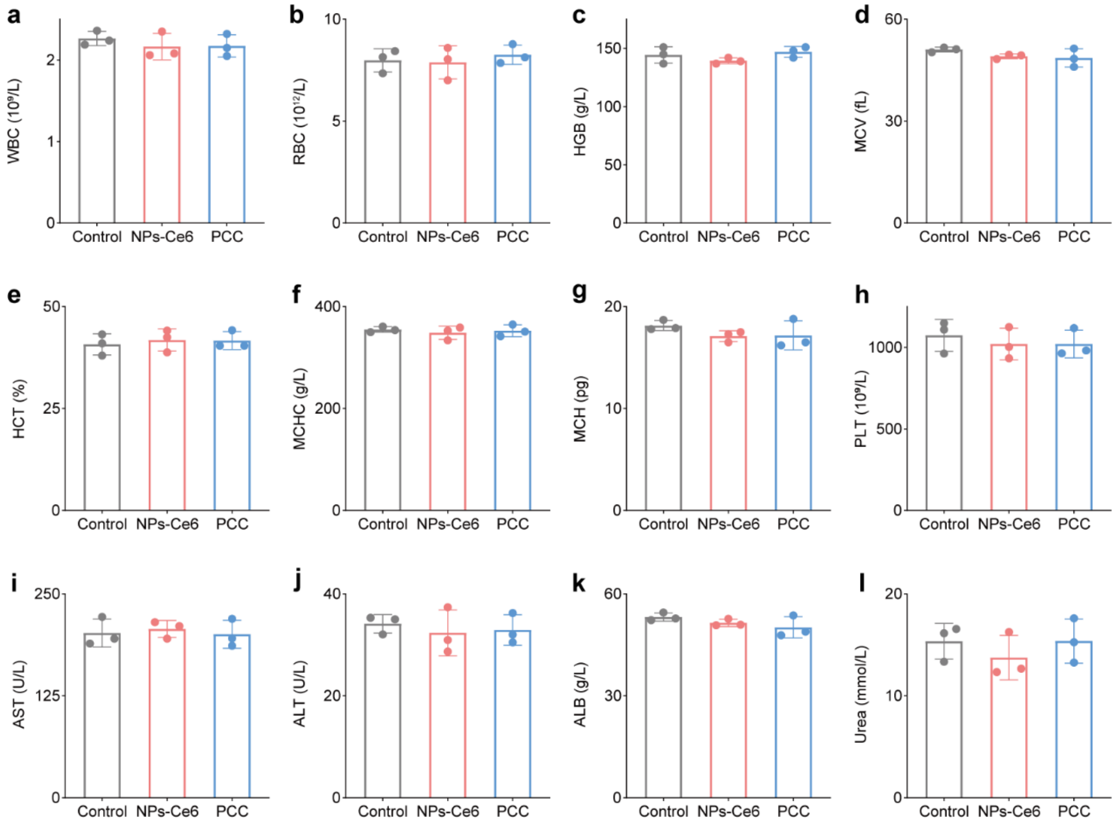

Figure S27. Blood hematological indexes (white blood cell, WBC; red blood cell, RBC; hemoglobin in blood, HGB; mean corpuscular volume, MCV; hematocrit, HCT; mean corpuscular hemoglobin concentration, MCHC; mean cell hemoglobin, MCH and platelet, PLT) and blood biochemistry analysis (aspartate transaminase, AST; alanine aminotransferase, ALT; albumin, ALB and urea) after systematic administration of NPs-Ce6 (500 µg/mL, 100 µL) and PCC (1×10^8 cell mL^{-1}, 100 µL) with PBS serving as control at day 7 (n = 3). Data are presented as mean values ± SD.

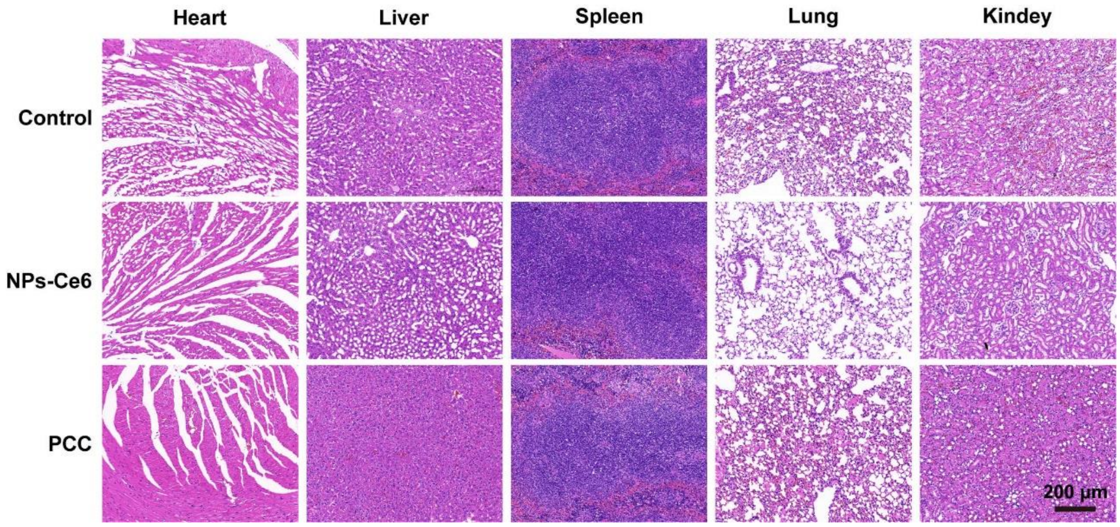

Figure S28. Representative H&E staining of the slices of mice organs harvested 7 d after systematic administration of NPs-Ce6 (500 µg/mL, 100 µL) and PCC (1×10^8 cell mL^{-1}, 100 µL) with PBS serving as control.

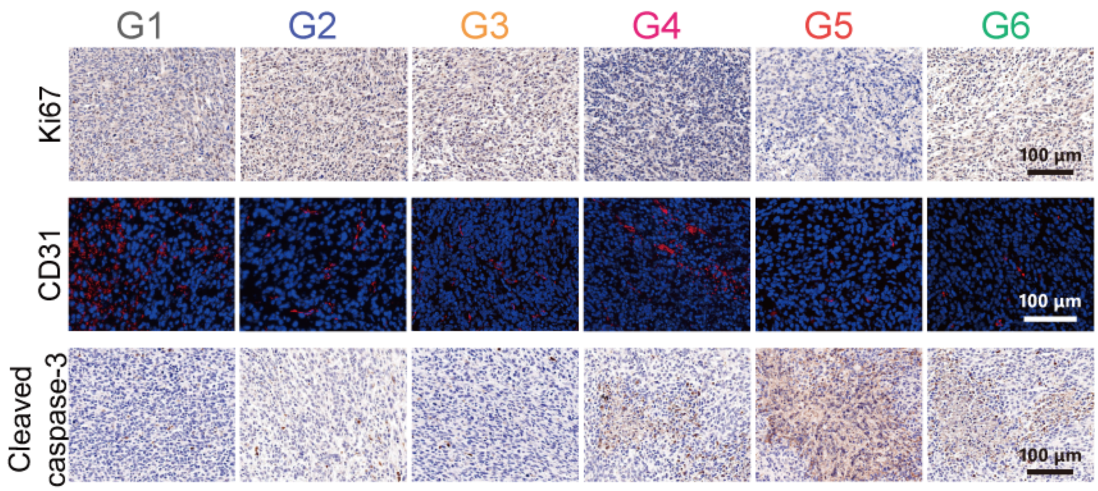

Figure S29. Representative Ki67 staining, CD31 immunohistochemical staining, and cleaved caspase-3 staining tumor sections from 4T1 tumor-bearing mice in different treatment groups. NPs-Ce6 concentration: 500 μg mL^{-1}, PCC concentration: 1 × 10^{8} cell mL^{-1}.

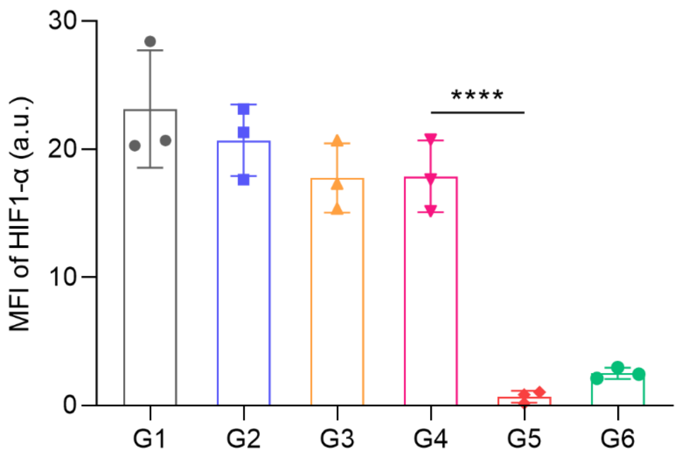

Figure S30. Mean fluorescence intensity of HIF-1α immunohistochemical staining images of 4T1-tumor sections from the tumor-bearing mice in different treatment groups (n = 3, NPs-Ce6 concentration: 500 μg mL^{-1}, PCC concentration: 1 × 10^{8} cell mL^{-1}). Data are presented as mean values ± SD. Statistical differences of p values were determined by One-way ANOVA. ****$p < 0.0001$.

Author contributions

W.F. and Y.C. conceived the study. Z.Y., X.S. and W.P. synthesized and characterized the nanoparticles. Z.Y., P.L. and X.J. characterized the cyanobacteria. Z.Y., W.P., C.W. and X.J. conducted in vitro and in vivo experiments. Z.Y., J.J., D.Y. and X.S. analyzed the data. Z.Y. wrote the original draft. X.S., W.P. and X.J. revised the paper. All authors discussed the experimental procedures and results.
